# Supplementary material for: Inferring effects of mutations on SARS-CoV-2 transmission from genomic surveillance data
Source: Nat Commun. 2025 Jan 7;16:441. doi: 10.1038/s41467-024-55593-0 (PMC11707167; doi:10.1038/s41467-024-55593-0)
Supplement: Supplementary file 1 — Supplementary Information [file 41467_2024_55593_MOESM1_ESM.pdf]

Supplementary Information

1. Summary

Here we discuss two main topics. First, we give a detailed introduction of our epidemiological model as well as a derivation of the estimator, (1) in the main text, and an important simplification of it. Second, we describe simulations of an outbreak and show that selection coefficients can be accurately recovered from simulation data even with relatively poor sampling.

2. Epidemiological model

1. Introduction

In epidemiology, the spread of infection can be modeled as a branching process where each infected individual (also referred to as a case) infects n additional individuals[1]. The distribution of n is often taken to be Poisson, but differences in the number of contacts with susceptible individuals, disease course within an individual, and other factors mean that the Poisson rate λ is not generally the same for all cases[2]. Below, we first follow ref.[2] to explore families of distributions for the number of new cases per infected individual. Next, we extend these models to consider multiple variants of the pathogen that differ in their spreading efficiency. We seek to characterize how the distribution of pathogen variant frequencies is expected to change over time, and how such data can be used to estimate the relative spreading efficiency of different variants.

2. Distributions for the number of infected individuals

As noted above, the basic distribution of the number of new cases n caused by one case in a susceptible population is Poisson,

$$P_{\mathrm{P}}(n|\lambda) = \frac{\lambda^n e^{-\lambda}}{n!}\,.$$

Typically we might take the Poisson rate λ to be R, the effective reproduction number, which is the expected number of cases directly caused by one case. In that case, the average number of cases following the Poisson distribution is

$$\langle n \rangle_{P_{\mathrm{P}}(n|R)} = \sum_{n=0}^{\infty} n P_{\mathrm{P}}(n|R) = R\,.$$

To account for variability in transmission dynamics, the basic Poisson distribution with a single rate R can be replaced with a continuous mixture of Poisson distributions, where the rate parameter λ follows a gamma distribution,

$$P_{\Gamma}(\lambda|\alpha,\beta) = \frac{\beta^{\alpha}}{\Gamma(\alpha)} \lambda^{\alpha-1} e^{-\beta\lambda}\,,$$

with shape parameter α and rate parameter β. The average value of λ is

$$\langle \lambda \rangle_{P_{\Gamma}(\lambda|\alpha,\beta)} = \frac{\alpha}{\beta}\,,$$

and its variance is

$$\left\langle \left(\lambda - \frac{\alpha}{\beta}\right)^2 \right\rangle_{P_{\Gamma}(\lambda|\alpha,\beta)} = \frac{\alpha}{\beta^2}\,.$$

In this context, it is natural to take $\alpha = k$ and $\beta = k/R$. With these choices, the gamma distribution reads

$$P_{\Gamma}(\lambda|k,R) = \frac{1}{\Gamma(k)} \left(\frac{k}{R}\right)^k \lambda^{k-1} e^{-k\lambda/R}\,. \qquad \textbf{(1)}$$

The parameter k is a dispersion parameter that determines how long-tailed the distribution is. The mean value of λ is always R, but when k is smaller its variance increases. In the limit that $k \to \infty$, we recover the pure Poisson distribution with rate $\lambda = R$. When $k = 1$, the distribution of the number of cases n is geometric,

$$\int_0^{\infty} d\lambda\, P_{\Gamma}(\lambda|k=1,R)\, P_{\mathrm{P}}(n|\lambda) = P_g(n|p) = (1-p)^n\, p\,,$$

where $p = 1/(1+R)$. For arbitrary values of $k > 0$, the number of cases follows a negative binomial distribution,

$$P_{\mathrm{NB}}(n|k,R) = \frac{\Gamma(k+n)}{n!\,\Gamma(k)} \left(\frac{k}{k+R}\right)^k \left(\frac{R}{k+R}\right)^n\,.$$

The standard parameters of the negative binomial distribution are r and p, which are set to k and $k/(k+R)$ in our parameterization above.

3. Dynamics for variant frequencies

Let us assume that there exist multiple variants of a pathogen, which are distinguished by an index a. The number of cases infected with variant a is n_a. We assume that different variants have slightly different transmission probabilities, so that $R_a = R(1+w_a)$, with $|w_a| \ll 1$. The term w_a is analogous to a selection coefficient in population genetics.

3.1. Dynamics of multiple cases infected by a single variant

First, let us assume that n individuals, each labeled by an index i, are all infected by the same variant of a pathogen. How many cases will be generated from these individuals? The number of new cases for all individuals is

$$n' = \sum_{i=1}^{n} n'_i ,$$

where the numbers of cases n'_i generated by individual i follows a negative binomial distribution. Because all individuals are infected by the same variant, the negative binomial parameter $p = k/(k+R)$ is the same for each of them. Then, assuming that all of the infection events are independent, it can be shown that the probability distribution for the total number of new cases n' also follows a negative binomial distribution with the same value of p, and with $r = nk$ (that is, the new r parameter value is the sum of the individual r parameter values). Thus, the distribution of n' is

$$P_{\mathrm{NB+}}(n'|k,R,n) = \frac{\Gamma(nk+n')}{n'!\,\Gamma(nk)} \left(\frac{k}{k+R}\right)^{nk} \left(\frac{R}{k+R}\right)^{n'} .$$

3.2. Dynamics for multiple cases infected by multiple variants

Let us extend the previous example to consider m variants of a pathogen. At the starting point, the number of individuals infected by a given variant a is n_a, with $a \in \{1,\ldots,m\}$. The fraction of cases infected by variant a is

$$y_a = \frac{n_a}{\sum_{b=1}^{m} n_b} .$$

Now, we would like to know how the fraction of individuals infected by each variant is expected to change with each round of infections. In other words, for variant a, we would like to compute

$$\langle y'_a \rangle = \left\langle \frac{n'_a}{\sum_{b=1}^{m} n'_b} \right\rangle = \sum_{\boldsymbol{n}'} \left(\prod_{b=1}^{m} P_{\mathrm{NB+}}(n'_b|k,R(1+w_b),n_b) \right) \frac{n'_a}{\sum_{c=1}^{m} n'_c}$$

where the outer sum is over all vectors $\boldsymbol{n}'$ with entries $\{n'_1, n'_2, \ldots\}$, and with $n'_b \geq 0$ for all b. Here, we have assumed that the n'_b's are independent across b.

To proceed, it is convenient to write the negative binomial distributions as mixtures of Poisson distributions (as indicated above), giving

$$\begin{aligned}
\langle y'_a \rangle &= \sum_{\boldsymbol{n}'} \left(\prod_{b=1}^{m} \int_0^\infty d\lambda_b \, P_\Gamma(\lambda_b|n_b k, R(1+w_b)) \, P_\mathrm{P}(n'_b|\lambda_b) \right) \frac{n'_a}{\sum_{c=1}^{m} n'_c} \\
&= \left(\prod_{b=1}^{m} \int_0^\infty d\lambda_b \, P_\Gamma(\lambda_b|n_b k, R(1+w_b)) \right) \sum_{\boldsymbol{n}'} \left(\prod_{b=1}^{m} P_\mathrm{P}(n'_b|\lambda_b) \right) \frac{n'_a}{\sum_{c=1}^{m} n'_c} .
\end{aligned}$$

Next, we use the fact that the sum of independent Poisson-distributed random variables is also Poisson with rate parameter equal to the sum of the individual rates, and that the distribution of independent Poisson random variables conditioned on their sum is multinomial, to write

$$\begin{aligned}
\langle y'_a \rangle &= \left(\prod_{b=1}^{m} \int_0^\infty d\lambda_b \, P_\Gamma(\lambda_b|n_b k, R(1+w_b)) \right) \sum_{n'=0}^{\infty} P_\mathrm{P}(n'|\lambda) \sum_{\boldsymbol{n}':\sum_{c=1}^{m} n'_c = n'} P_\mathrm{M}\left(\boldsymbol{n}' \middle| n', \frac{\boldsymbol{\lambda}}{\lambda}\right) \frac{n'_a}{n'} \\
&= \left(\prod_{b=1}^{m} \int_0^\infty d\lambda_b \, P_\Gamma(\lambda_b|n_b k, R(1+w_b)) \right) \sum_{n'=0}^{\infty} P_\mathrm{P}(n'|\lambda) \frac{\lambda_a}{\lambda} \\
&= \left(\prod_{b=1}^{m} \int_0^\infty d\lambda_b \, P_\Gamma(\lambda_b|n_b k, R(1+w_b)) \right) \frac{\lambda_a}{\lambda} .
\end{aligned}$$

Here $\boldsymbol{\lambda}$ is a vector with entries $\{\lambda_1, \lambda_2, \ldots\}$, and we have also introduced $\sum_a \lambda_a = \lambda$. Note also that the outer sum on the first line is over all vectors $\boldsymbol{n}'$ whose (non-negative) entries sum to n'.

Computing the remaining integrals exactly is challenging, largely because the Gamma distributions have different rate parameters. To address this, next we will expand our expression to first order in the w_a, since these are assumed to be small parameters. Referring back to Eq. (1), the expansion gives

$$\begin{aligned}\langle y_a' \rangle &= \left(\prod_{b=1}^{m} \int_0^\infty d\lambda_b \, P_\Gamma\left(\lambda_b | n_b k, R\right) \left[1 - k w_b \left(n_b - \frac{\lambda_b}{R}\right)\right]\right) \frac{\lambda_a}{\lambda} + \mathcal{O}\left(w^2\right) \\ &= \left(\prod_{b=1}^{m} \int_0^\infty d\lambda_b \, P_\Gamma\left(\lambda_b | n_b k, R\right)\right) \left[1 - \sum_{c=1}^{m} k w_c \left(n_c - \frac{\lambda_c}{R}\right)\right] \frac{\lambda_a}{\lambda} + \mathcal{O}\left(w^2\right) .\end{aligned}$$

Next we change variables to $\{\lambda, q_1 = \lambda_1/\lambda, q_2 = \lambda_2/\lambda, \ldots, q_{m-1} = \lambda_{m-1}/\lambda\}$, because the distribution of the sum of gamma-distributed random variables, λ, with the same rate parameter and the ratios of the individual variables to the total (λ_a/λ) follow independent gamma and Dirichlet distributions[3]. The mth ratio $q_m = 1 - \sum_{a=1}^{m-1} q_a$ by conservation. By convention we will also set $w_m = 0$, which can be thought of as normalizing the value of R relative to a reference genotype. The transformation then gives

$$\begin{aligned}\langle y_a' \rangle &= \int_0^\infty d\lambda \, P_\Gamma\left(\lambda | nk, R\right) \left(\prod_{b=1}^{m-1} \int dq_b\right) P_\mathrm{D}\left(\boldsymbol{q} | \boldsymbol{n}k\right) \left[1 - \sum_{c=1}^{m} k w_c \left(n_c - \frac{\lambda q_c}{R}\right)\right] q_a \\ &= \left(\prod_{b=1}^{m-1} \int dq_b\right) P_\mathrm{D}\left(\boldsymbol{q} | \boldsymbol{n}k\right) \left[1 - \sum_{c=1}^{m} k w_c \left(n_c - n q_c\right)\right] q_a \\ &= \left(1 - k \sum_{c=1}^{m} n_c w_c\right) y_a + \left(\prod_{b=1}^{m-1} \int dq_b\right) P_\mathrm{D}\left(\boldsymbol{q} | \boldsymbol{n}k\right) nk \left(\sum_{c \neq a} w_c q_c q_a + w_a q_a^2\right) \\ &= \left(1 - nk \sum_{b=1}^{m} w_b y_b\right) y_a + \frac{nk}{nk+1} \left[nk \sum_{b \neq a} w_b y_a y_b + w_a \left(nk y_a^2 + y_a\right)\right] \\ &= y_a + \frac{nk}{nk+1} y_a \left(w_a - \sum_{b=1}^{m} w_b y_b\right) .\end{aligned}$$

In the expressions above $P_\mathrm{D}(\boldsymbol{q}|\boldsymbol{\alpha})$ is the Dirichlet distribution, with concentration parameters $\boldsymbol{\alpha}$ given by $\boldsymbol{n}k$ in our case. Note that if $w_m \neq 0$, the last line should instead read

$$\langle y_a' \rangle = y_a + \frac{nk}{nk+1} y_a \left(w_a - w_m - \sum_{b=1}^{m} w_b y_b\right) .$$

Thus, we obtain (with $w_m = 0$)

$$\langle y_a' - y_a \rangle = \langle \Delta y_a \rangle = \frac{nk}{nk+1} y_a \left(w_a - \sum_{b=1}^{m} w_b y_b\right) .$$

Following a similar approach, we can compute the second moments. First, we consider

$$\begin{aligned}\left\langle (y_a')^2 \right\rangle &= \left\langle \left(\frac{n_a'}{\sum_{b=1}^{m} n_b'}\right)^2 \right\rangle \\ &= \left(\prod_{b=1}^{m} \int_0^\infty d\lambda_b \, P_\Gamma\left(\lambda_b | n_b k, R(1+w_b)\right)\right) \sum_{n'=0}^{\infty} P_\mathrm{P}\left(n' | \lambda\right) \sum_{\boldsymbol{n}' : \sum_{c=1}^{m} n_c' = n'} P_\mathrm{M}\left(\boldsymbol{n}' \middle| n', \frac{\boldsymbol{\lambda}}{\lambda}\right) \left(\frac{n_a'}{n'}\right)^2 \\ &= \left(\prod_{b=1}^{m} \int_0^\infty d\lambda_b \, P_\Gamma\left(\lambda_b | n_b k, R(1+w_b)\right)\right) \sum_{n'=0}^{\infty} P_\mathrm{P}\left(n' | \lambda\right) \left[\left(\frac{\lambda_a}{\lambda}\right)^2 + \frac{1}{n'} \frac{\lambda_a}{\lambda} \left(1 - \frac{\lambda_a}{\lambda}\right)\right] \\ &\approx \left(\prod_{b=1}^{m} \int_0^\infty d\lambda_b \, P_\Gamma\left(\lambda_b | n_b k, R\right)\right) \left[1 - \sum_{c=1}^{m} k w_c \left(n_c - \frac{\lambda_c}{R}\right)\right] \left[\left(\frac{\lambda_a}{\lambda}\right)^2 + \frac{1}{\lambda} \frac{\lambda_a}{\lambda} \left(1 - \frac{\lambda_a}{\lambda}\right)\right] \\ &= \int_0^\infty d\lambda \, P_\Gamma\left(\lambda | nk, R\right) \left(\prod_{b=1}^{m-1} \int dq_b\right) P_\mathrm{D}\left(\boldsymbol{q} | \boldsymbol{n}k\right) \left[1 - \sum_{c=1}^{m} k w_c \left(n_c - \frac{\lambda q_c}{R}\right)\right] \left[q_a^2 + \frac{q_a(1-q_a)}{\lambda}\right] .\end{aligned}$$

In going from the third to the fourth line above, we have made the approximation that

$$\left\langle \frac{1}{n'} \right\rangle_{P_{\mathrm{P}}(n'|\lambda)} \approx \frac{1}{\lambda},$$

which is valid for $\lambda \gtrsim 1$. Similarly,

$$\begin{aligned}
\langle y'_a y'_b \rangle &= \left\langle \frac{n'_a n'_b}{\left(\sum_{c=1}^{m} n'_c\right)^2} \right\rangle \\
&= \int_0^\infty \left(\prod_{c=1}^{m} d\lambda_c \, P_\Gamma\left(\lambda_c | n_c k, R(1+w_c)\right) \right) \sum_{n'=0}^{\infty} P_{\mathrm{P}}\left(n'|\lambda\right) \left(1 - \frac{1}{n'}\right) \frac{\lambda_a \lambda_b}{\lambda^2} \\
&\approx \int_0^\infty \left(\prod_{c=1}^{m} d\lambda_c \, P_\Gamma\left(\lambda_c | n_c k, R\right) \right) \left[1 - \sum_{d=1}^{m} k w_d \left(n_d - \frac{\lambda_d}{R}\right)\right] \left(1 - \frac{1}{\lambda}\right) \frac{\lambda_a \lambda_b}{\lambda^2} \\
&= \int_0^\infty d\lambda \, P_\Gamma\left(\lambda | nk, R\right) \left(\prod_{c=1}^{m-1} \int dq_c \right) P_{\mathrm{D}}\left(\boldsymbol{q} | \boldsymbol{n}k\right) \left[1 - \sum_{d=1}^{m} k w_d \left(n_d - \frac{\lambda q_d}{R}\right)\right] \left(1 - \frac{1}{\lambda}\right) q_a q_b \,.
\end{aligned}$$

Simplifying the expressions above is tedious but straightforward. The following results are helpful:

$$\begin{aligned}
\int_0^\infty d\lambda \, P_\Gamma\left(\lambda | nk, R\right) \lambda &= nR, \\
\int_0^\infty d\lambda \, P_\Gamma\left(\lambda | nk, R\right) \frac{1}{\lambda} &= \frac{k/R}{nk-1}, \\
\left(\prod_{c=1}^{m-1} \int dq_c \right) P_{\mathrm{D}}\left(\boldsymbol{q} | \boldsymbol{n}k\right) q_a q_b &= \frac{nk}{nk+1} y_a y_b, \\
\left(\prod_{b=1}^{m-1} \int dq_b \right) P_{\mathrm{D}}\left(\boldsymbol{q} | \boldsymbol{n}k\right) q_a^2 &= y_a^2 + \frac{y_a(1-y_a)}{nk+1} = \frac{nk}{nk+1} y_a^2 + \frac{1}{nk+1} y_a, \\
\left(\prod_{c=1}^{m-1} \int dq_c \right) P_{\mathrm{D}}\left(\boldsymbol{q} | \boldsymbol{n}k\right) q_a^2 q_b &= \left(y_a^2 + \frac{y_a(1-y_a)}{nk+1} \right) \frac{nk}{nk+2} y_b, \\
\left(\prod_{b=1}^{m-1} \int dq_b \right) P_{\mathrm{D}}\left(\boldsymbol{q} | \boldsymbol{n}k\right) q_a^3 &= \left(y_a^2 + \frac{y_a(1-y_a)}{nk+1} \right) \frac{nk y_a + 2}{nk+2}.
\end{aligned}$$

Here we have frequently used $n_a = n y_a$ to simplify expressions.

With the above results, simplifying expressions for the second moments, we finally find

$$\left\langle (\Delta y_a)^2 \right\rangle = \left[\frac{1}{nk+1} + \frac{nk}{nk+1} \frac{k/R}{nk-1} \right] y_a \left(1 - y_a\right) + \mathcal{O}\left(1/n^2\right),$$

and

$$\langle \Delta y_a \Delta y_b \rangle = - \left[\frac{1}{nk+1} + \frac{nk}{nk+1} \frac{k/R}{nk-1} \right] y_a y_b + \mathcal{O}\left(1/n^2\right),$$

where we have assumed that the w_a are $\mathcal{O}(1/n)$, as in the Wright-Fisher model with weak selection. We have thus found that the first and second moments of frequency changes in our multi-variant epidemiological model have the same frequency dependence as those in the multispecies Wright-Fisher model, but with different scaling. The first moment ('drift') is multiplied by a factor of $nk/(nk+1)$, and the second moment ('diffusion') by

$$\frac{1}{nk+1} + \frac{nk}{nk+1} \frac{k/R}{nk-1}.$$

These prefactors match with the Wright-Fisher model exactly when $k \to \infty$ (i.e., a pure Poisson distribution for the number of new cases per infected individual) and $R = 1$.

4. Derivation of the selection coefficient estimator

The derivation in this section closely follows that given in ref.[4]. It is well known that a WF process can be approximated by a continuous-time continuous-frequency diffusion process in the large n limit. In the continuous-time limit the time variable t has units of n generations, with one generation in discrete time taking $\tau = 1/n$ continuous time units. The selection coefficients w_a are assumed to scale with n such that $w_a = \tilde{w}_a/n$, where $\tilde{w}_a$ is a parameter independent of the population size n. In the limit of large population size, our generalized super-spreading model can, like the WF process, be approximated by a diffusion process, where the transition probability density ϕ is the solution to the Fokker-Planck equation

$$\frac{\partial \phi}{\partial t} = \left[-\sum_{a=1}^{M} \frac{\partial}{\partial x_a} \boldsymbol{d}(\boldsymbol{y}(t)) + \sum_{a=1}^{M}\sum_{b=1}^{M} \frac{\partial}{\partial y_a}\frac{\partial}{\partial y_b} C_{ab}(\boldsymbol{y}(t)) \right] \phi \,,$$

where M is the number of distinct genotypes, $\boldsymbol{y}$ is the genotype frequency vector, $\boldsymbol{d}$ is the drift vector, and C is the diffusion matrix. Here we ignore recombination and mutation, since these are comparatively small and therefore unlikely to significantly affect estimates of changes in viral transmission (though these can be included and the solution remains tractable). The drift and diffusion have entries given by,

$$\begin{aligned}
\tilde{d}_a(\boldsymbol{y}(t)) &= \lim_{n\to\infty} n \left\langle \Delta y_a \right\rangle \\
&= \lim_{n\to\infty} \frac{nk}{nk+1} y_a(t) \left(w_a - \sum_{b=1}^{M} w_b y_b(t) \right) \\
&= y_a(t) \left(\tilde{w}_a - \sum_{b=1}^{M} \tilde{w}_b y_b(t) \right) , \\
\tilde{C}_{ab}(\boldsymbol{y}(t)) &= \frac{1}{2} \lim_{n\to\infty} n \left\langle \Delta y_a \Delta y_b \right\rangle \\
&= \frac{1}{2}\left[\frac{1}{k} + \frac{1}{R} \right] \begin{cases} y_a(t)\,(1-y_a(t)) & a = b \\ -y_a(t) y_b(t) & a \neq b \,. \end{cases}
\end{aligned}$$

For genotype frequencies observed at times t and $t+\tau\Delta t$ (i.e., over Δt generations), and for small $\tau\Delta t$, the Fokker-Planck equation can be converted into a path integral approximation for the transition probability density (see ref.[4] for a rigorous derivation)

$$\begin{aligned}
&\phi\left(\boldsymbol{y}(t+\tau\Delta t) | \boldsymbol{y}(t)\right) \\
&\approx \frac{\exp\left\{ -\frac{4n}{\Delta t} \sum_{a=1}^{M}\sum_{b=1}^{M} \left[y_a(t+\tau\Delta t) - y_a(t) - \tilde{d}_a(\boldsymbol{y}(t))\tau\Delta t \right] \left(\tilde{C}^{-1}(y_a(t)) \right)_{ab} \left[y_b(t+\tau\Delta t) - y_b(t) - \tilde{d}_b(\boldsymbol{y}(t))\tau\Delta t \right] \right\}}{(4\pi\tau\Delta t)^{M/2} \sqrt{\det\left(\tilde{C}(\boldsymbol{y}(t))\right)}} \,.
\end{aligned}$$

From this result, and recalling $\tau = 1/n$, the transition probability from time t_m to t_{m+1} of the original branching process (for large $n/\Delta t$) can be approximated by

$$\begin{aligned}
&P\left(\boldsymbol{y}(t_{m+1}) | \boldsymbol{y}(t_m)\right) \\
&\approx \phi\left(\boldsymbol{y}(t_{m+1}) | \boldsymbol{y}(t_m)\right) \prod_{a=1}^{M} dy_a(t_{m+1}) \\
&= \frac{\exp\left\{ -\frac{n}{2} \sum_{a=1}^{M}\sum_{b=1}^{M} \left[\frac{y_a(t_{m+1})-y_a(t_m)}{\Delta t_m} - d_a(\boldsymbol{y}(t_m)) \right] \left(C^{-1}(y_a(t_m)) \right)_{ab} \left[\frac{y_b(t_{m+1})-y_b(t_m)}{\Delta t_m} - d_b(\boldsymbol{y}(t_m)) \right] \right\}}{(2\pi\Delta t_m/n)^{M/2} \sqrt{\det\left(C(\boldsymbol{y}(t_m))\right)}} \prod_{a=1}^{M} dy_a(t_{m+1}) \,,
\end{aligned}$$

where we write the re-scaled drift vector as $d_a = \tilde{d}_a \tau$, the re-scaled diffusion matrix as $C_{ab} = 2\tilde{C}_{ab}$, and $\Delta t_m = t_{m+1} - t_m$. Since we aim to infer selection coefficients for the SNVs, it is more convenient to work with the allele frequencies x_i instead of the genotype frequencies y_a. The allele frequency at site i is given by

$$x_i(t_m) = \sum_{a=1}^{M} g_i^a y_a(t_m) \,,$$

where g_i^a is a 1 if there there is a mutant allele at site i on genome a and zero if there is not. Similarly, if the selection coefficient for the genotype a is w_a and the allele level selection coefficient for allele j is s_j, then they are related by:

$$w_a = \sum_{j=1}^{L} g_j^a s_j \ ,$$

where L is the length of the genome.

The allele level drift and diffusion terms will be linear combinations of the genotype level drift and diffusion, just as with the frequencies and the selection coefficients. The drift vector for the allele frequencies can be transformed by

$$\begin{aligned} d_i\left(\boldsymbol{x}(t_m)\right) &= \sum_{a=1}^{M} g_i^a d_a\left(\boldsymbol{y}(t_m)\right) \\ &= \sum_{a=1}^{M} g_i^a y_a(t_m)\left(w_a - \sum_{b=1}^{M} w_b y_b(t_m)\right) \\ &= x_i(t_m)(1-x_i(t_m))s_i + \sum_{j=1, j\neq i}^{L} \left(x_{ij}(t_m) - x_i(t_m)x_j(t_m)\right) s_j \ . \end{aligned}$$

This can be used, along with the transition probability density for genomes, in order to find an approximation for the mutant allele transition probability density:

$$\begin{aligned} &P\left(\boldsymbol{x}(t_{m+1})|\boldsymbol{x}(t_m)\right) \\ &\approx \frac{\exp\left\{-\frac{n}{2}\sum_{i=1}^{L}\sum_{j=1}^{L}\left[\frac{x_i(t_{m+1})-x_i(t_m)}{\Delta t_m} - d_i(\boldsymbol{x}(t_m))\right]\left(C^{-1}(\boldsymbol{x}(t_m))\right)_{ij}\left[\frac{x_j(t_{m+1})-x_j(t_m)}{\Delta t_m} - d_j(\boldsymbol{x}(t_m))\right]\right\}}{(2\pi\Delta t_m/n)^{L/2}\sqrt{\det\left(C(\boldsymbol{x}(t_m))\right)}} \prod_{i=1}^{L} dx_i(t_{m+1}) \ , \end{aligned}$$

where here the diffusion C is derived similarly to the drift $\boldsymbol{d}$ and has entries

$$C_{ij}(\boldsymbol{x}(t_m)) = \left[\frac{1}{k} + \frac{1}{R}\right]\left(x_{ij}(t_m) - x_i(t_m)x_j(t_m)\right) .$$

A path integral then gives the probability of observing a trajectory of allele frequencies $(\boldsymbol{x}(t_1), \boldsymbol{x}(t_2), ..., \boldsymbol{x}(t_{T-1}))$, and is given by

$$P\left(\left(\boldsymbol{x}(t_m)\right)_{m=1}^{T} \left|\boldsymbol{x}(t_0)\right.\right) = \prod_{m=0}^{T-1} P\left(\boldsymbol{x}(t_{m+1})|\boldsymbol{x}(t_m)\right) .$$

Bayesian analysis can then be used to show that the posterior probability of the selection coefficients $\boldsymbol{s} = (s_1, s_2, ..., s_L)$ given an observed frequency path $\boldsymbol{x}(t_0), \boldsymbol{x}(t_1), ..., \boldsymbol{x}(t_{T-1})$ is

$$P\left(\boldsymbol{s}\middle|\left(\boldsymbol{x}(t_m)\right)_{m=0}^{T}\right) \propto P\left(\left(\boldsymbol{x}(t_m)\right)_{m=1}^{T} \left|\boldsymbol{x}(t_0)\right.\right) \times P_{\text{Prior}}(\boldsymbol{s}) \,, \tag{2}$$

where we use a Gaussian prior distribution with zero mean and adjustable covariance determined by the parameter γ, which is the precision.

For the inferred coefficients, we take those that maximize the posterior probability. They can be analytically found by a simple application of the Euler-Lagrange equations to (2), or the equivalent expression at the genotype level, and are given by

$$\hat{\boldsymbol{s}} = \left[\gamma I + \sum_m n \frac{k^2 R^2}{(R+k)^2} C(t_m)\right]^{-1} \left[\sum_m \frac{nkR}{k+R}\left(\Delta\boldsymbol{x}(t_m)\right)\right] . \tag{3}$$

5. Extension to multiple regions

In the SARS-CoV-2 pandemic, and in real disease outbreaks in general, there are frequently multiple different outbreaks in different regions that develop largely or entirely independently of one another. In order to find the best estimate for the selection coefficients using the data from multiple regions, the estimator can be generalized to find the maximum a posteriori estimate for the selection coefficients given the time series of allele frequencies in each of the regions. If the probability for a specific

path in a specific region r is given by $P\left((\boldsymbol{x}_r(t_{r,m}))_{m=1}^{T_r} | \boldsymbol{x}_r(t_{r,0})\right)$, where x_r is the allele frequency vector in region r, then the joint probability of the specific paths in all of the regions is simply the product of the individual region probabilities:

$$P\left((\boldsymbol{x}_1(t_{1,m}))_{m=1}^{T_1},\ldots,(\boldsymbol{x}_Q(t_{Q,m}))_{m=1}^{T_Q} \big| \{\boldsymbol{x}_r(t_{r,0})\}_{r=1}^{Q}\right) = \prod_{r=1}^{Q} P\left((\boldsymbol{x}_r(t_{r,m}))_{m=1}^{T_r} | \boldsymbol{x}_r(t_{r,0})\right),$$

where Q is the number of different regions. Since this is a product of exponential functions, the log posterior will be the sum of the exponents and the regularization. This can be maximized with respect to the selection coefficient vector $\boldsymbol{s}$ as before and leads to the estimator:

$$\hat{\boldsymbol{s}} = \left[\gamma I + \sum_r \sum_{t_{r,m}} \frac{n_r k_r^2 R_r^2}{(k_r+R_r)^2} C_r(t_{r,m})\right]^{-1} \left[\sum_r \sum_{t_{r,m}} \frac{k_r n_r R_r}{k_r+R_r} \Delta \boldsymbol{x}_r(t_{r,m})\right]. \quad \textbf{(4)}$$

6. Simplification of the estimator

In real outbreaks the parameters k, R, and n are in general time-varying. In our simulations as well, R and n are time-varying (and k can be constant or time-varying). In order to accurately infer the selection coefficients according to Eq. (3) or Eq. (4), it would seem that we need to accurately infer the values of k, R, and N at every point in the time series. In practice, this would be extremely difficult. For general discussion about the effective reproduction number R and the basic reproduction number R_t as well as some attempts to infer this, see refs.[5–9]. In order to get an accurate estimate for k it is necessary to have pervasive contact tracing, so that the negative binomial distribution is well sampled, and there are other difficulties in inferring k as well[10–12]. Lastly, it can be difficult to estimate the number of new infections due to multiple factors, including the difference between the population that gets tested and the population that does not, test result inaccuracies, and delays between symptom onset, testing, and reporting.

We propose an alternative that lets us avoid these complications. The prefactor $nkR/(R+k)$, multiplies both the numerator and the denominator. Therefore, the only effect of the prefactor is to weight time points more heavily if the population size, the dispersion parameter, or the basic reproduction number, is larger. This makes sense in theory, because a larger n or k implies that there is less noise and the trajectories are more deterministic, while a larger R means that there are more new infections per generation and thus more data to use to infer the selection coefficients. This does hold with perfect information, that is, if all infected individuals are sampled at every time point. However, in practice, finite sampling is the source of significantly more noise than that due to a time-varying population size or dispersion, so weighting the time points based upon n, k, or R in fact leads to worse inference than assuming the parameters are constant in time and thus weighting the time points equally. However, in the special and unrealistic case of perfect sampling, using the actual parameters does lead to better inference than using constant parameters (see **Supplementary Fig. 11**). If the time points are weighted equally, then, provided that the regularization γ is scaled appropriately (and in general it must be determined by separate means, discussed below), the prefactors in the numerator and denominator cancel, and the estimator is independent of n, k, and R. Defining $\gamma' = \gamma nkR/(k+R)$ and $\bar{C}$ by

$$C = \left[\frac{nkR}{k+R}\right]\bar{C},$$

so that

$$\bar{C}_{ij} = \begin{cases} x_{ij}(t_m) - x_i(t_m)x_j(t_m) & i \neq j \\ x_i(t_m)\left(1 - x_i(t_m)\right) & i = j \end{cases},$$

Eqs. (3) and (4) for the selection coefficients become, respectively

$$\hat{\boldsymbol{s}} = \left[\gamma' I + \sum_{t_m} \bar{C}(t_m)\right]^{-1} \left[\sum_{t_m} \Delta \boldsymbol{x}(t_m)\right],$$

$$\hat{\boldsymbol{s}} = \left[\gamma' I + \sum_r \sum_{t_{r,m}} \bar{C}_r(t_{r,m})\right]^{-1} \left[\sum_r \sum_{t_{r,m}} \Delta \boldsymbol{x}_r(t_{r,m})\right],$$

which are the same as the MPL estimators for the Wright-Fisher model except for the absence of a mutation term[4].

7. Covariance of the inferred selection coefficients

Since the posterior given in (2) is a Gaussian distribution for the selection coefficients, the covariance matrix of the inferred selection coefficients can be easily found. For any Gaussian distributed random vector $\boldsymbol{z}$, the inverse of the covariance can be

calculated as the second derivative with respect to $\boldsymbol{z}$ of the negative log of the probability density function. That is, if we define

$$\begin{aligned}J &= -\ln\left[P\left(\boldsymbol{s}\middle|\,(\boldsymbol{x}(t_m))_{m=0}^{T}\right)\right] \\ &= \frac{1}{2}\left[\gamma\boldsymbol{s}^2 + \sum_{m=0}^{T-1} n\left[\boldsymbol{x}(t_{m+1}) - \boldsymbol{x}(t_m) - \boldsymbol{d}(\boldsymbol{x}(t_m))\right]^T C^{-1}(\boldsymbol{x}(t_m))\left[\boldsymbol{x}(t_{m+1}) - \boldsymbol{x}(t_m) - \boldsymbol{d}(\boldsymbol{x}(t_m))\right]\right. \\ &\qquad \left. + \sum_{m=0}^{T-1}\left(L\ln\left(\frac{2\pi}{n}\right) + \ln(\det C)\right)\right],\end{aligned}$$

then the inverse of the covariance matrix of the parameters is given by the second derivative of J with respect to $\boldsymbol{s}$. The first derivative of J with respect to $\boldsymbol{s}$ gives

$$\frac{\partial J}{\partial \boldsymbol{s}} = \gamma\boldsymbol{s} - \sum_{m=0}^{T-1}\frac{nkR}{k+R}CC^{-1}\left[\boldsymbol{x}(t_{m+1}) - \boldsymbol{x}(t_m) - \boldsymbol{d}(\boldsymbol{x}(t_m))\right].$$

The second derivative, which is the inverse of the covariance of the selection coefficients $\boldsymbol{s}$, is

$$\frac{\partial^2 J}{\partial \boldsymbol{s}\partial \boldsymbol{s}^T} = \gamma + \sum_{m=0}^{T-1}\frac{nk^2R^2}{(k+R)^2}C(\boldsymbol{x}(t_m)).$$

This implies that the covariance of the inferred coefficients is given by

$$\Sigma = \left[\gamma I + \sum_{m=0}^{T-1}\frac{nk^2R^2}{(k+R)^2}C(\boldsymbol{x}(t_m))\right]^{-1}.$$

Using the definitions of γ' and $\bar{C}$ given above, in the case where the parameters n, k, and R are constant, this reduces to

$$\Sigma = \frac{k+R}{nkR}\left[\gamma' I + \sum_{m=0}^{T-1} C(\boldsymbol{x}(t_m))\right]^{-1}. \tag{5}$$

Since $(k+R)/nkR$ is a decreasing function of k, this implies that the theoretical covariance decreases as the dispersion k becomes larger. **Supplementary Fig. 14a** shows the theoretical uncertainty in the selection coefficients with the largest magnitudes that we infer from SARS-CoV-2 data. Because the theoretical uncertainties do not account for finite sampling, these error bars tend to be fairly small. To obtain more realistic error bars, we also performed bootstrap resampling of the data, where multiple regions were also omitted from the analysis at random (**Supplementary Fig. 14b**).

8. Covariance of inferred selection coefficients for a group of fully linked sites

The above analysis can be used to quantify the covariance between inferred coefficients for a group of SNVs that are fully linked, meaning that all of the SNVs in the group appear together on every sequence on which one of the SNVs appear. This is useful because it provides an estimate for the maximum covariance between linked SNVs. An analytical result is presented only for the special case where all of the SNVs under consideration are fully linked, though simulations indicate that the maximum value is not strongly dependent on other SNVs that are partially linked to the main group. The covariance matrix at any time for a group of fully linked SNVs has (i,j)th element given by $(C(t_m))_{ij} = \left[\frac{1}{k} + \frac{1}{R}\right] x_i(t_m)(1 - x_i(t_m))$ for any (i,j), since the frequencies $x_i(t_m)$ for all of the SNVs are identical. This implies that the second term in (5) is a matrix with every entry identical. If we define the elements of the matrix

$$\sum_{m=0}^{T-1}\frac{nk^2R^2}{(k+R)^2}C_{ij}(\boldsymbol{x}(t_m)) \equiv \alpha,$$

the vector $\boldsymbol{u}$ as the vector of all 1's, and use the notation $(\cdot)^{\mathrm{T}}$ to denote transpose, then the covariance of the inferred coefficients can be written as

$$\Sigma_{\text{linked}} = \left[\gamma I + \alpha\boldsymbol{u}\boldsymbol{u}^{\mathrm{T}}\right]^{-1}.$$

Because of the simplicity of this form of the matrix, the inversion can be carried out explicitly using the Sherman-Morrison formula, which for an $n \times n$ matrix gives

$$\begin{aligned}\Sigma_{\text{linked}} &= \frac{1}{\gamma}I - \frac{\alpha \frac{1}{\gamma^2} I \boldsymbol{u}\boldsymbol{u}^{\mathrm{T}} I}{1 + \alpha \boldsymbol{u}^{\mathrm{T}} I \boldsymbol{u} \frac{1}{\gamma}} \\ &= \frac{1}{\gamma}I - \frac{1}{\frac{\gamma^2}{\alpha} + \gamma n} \boldsymbol{u}\boldsymbol{u}^{\mathrm{T}} .\end{aligned}$$

From this the correlation matrix can be easily calculated, and the off-diagonal elements represent the maximum correlation between n SNVs that are fully linked to one another. The off diagonal elements of the correlation matrix are given by

$$\rho_{i,j} = \frac{1}{1 - n - \frac{\gamma}{\alpha}} .$$

We analyzed sets of strongly linked mutations in the Alpha, Delta, and Omicron variants to test our ability to distinguish the independent selective effects of individual mutations. **Supplementary Figure 15** shows that, while many inferred selection coefficients are naturally correlated, this correlation is far from complete. Only in rare circumstances (e.g., the three nucleotide mutations comprising N:D3L in Alpha) are SNVs so strongly linked that their effects cannot be at least partially disentangled.

3. Simulations

We tested the inference using simulations of disease spread. Specifically, we ran super-spreader simulations based on the model described above, which is an analog of the Wright-Fisher model where the sampling distribution for the number of new infections per infected individual is drawn from a negative binomial distribution instead of a pure Poisson distribution.

1. Description of simulations

We simulated disease spread as a branching process in which the number of individuals infected per currently infected individual is drawn from a negative binomial distribution whose shape is determined by the basic reproduction number R_0 (or the reproduction number, R, in a population that is not totally susceptible) and the dispersion parameter k. Because we sample in this way, the population size is not constant. However, if the population size is too small, then the population is extremely likely to die off stochastically, and if the population size is too large, then sampling from the negative binomial becomes too computationally expensive. In order to avoid both of these problems, once the population size is large enough R is adaptively adjusted so that the average reproduction number for the entire population will remain near 1, and the population size will oscillate around a fixed value. An explicit time-varying population size can also be used as input, and R will be adaptively adjusted to remain near the given curve. Constant values can be used for the dispersion k or k can vary as a function of time, perhaps representing different degrees of social distancing or lockdown measures at different times. Since different interventions implemented to prevent the spread of disease would likely affect the shape of the distribution of the number of individuals infected by a single infected individual, time-varying values for k and R can be used to reflect these effects.

2. Inference

The simulations are run for a number of generations and genomes are sampled from the population of infected individuals at different times using a multinomial sampling distribution. This sampled time series is then used to infer the selection coefficients using (3). Alternatively, multiple simulations can be run and the joint inference of the selection coefficients can be made using (4). We find that, given good enough sampling, a long enough time series, and sampling that occurs at a sufficient number of times, the selection coefficients can be inferred very accurately (**Fig. 1**). The quality of inference is significantly improved if multiple simulations are combined and if mutated sites show up in more than one of the simulations, even under less than ideal sampling conditions. Beneficial coefficients are typically inferred more accurately than deleterious ones, likely because deleterious SNVs frequently die off and therefore there is less data to use for inference.

The inference is robust to shortening the time-series or lowering the number of samples taken per generation, though obviously if either of these conditions is too extreme (or worse, both), the inference starts to break down. The negative effects of a short time-series or poor sampling can be somewhat made up for by using multiple simulations, which is analogous to using data from outbreaks in multiple regions. In addition, the diffusion approximation is only valid in the large n limit. However, we tested the inference for small population sizes and found that inference is accurate even if the population of newly infected individuals per serial interval is as low as a few hundred (**Supplementary Fig. 1**).

Supplementary References

1. Diekmann, O. & Heesterbeek, J. A. P. *Mathematical epidemiology of infectious diseases: Model building, analysis and interpretation*, vol. 5 (John Wiley & Sons, 2000).
2. Lloyd-Smith, J. O., Schreiber, S. J., Kopp, P. E. & Getz, W. M. Superspreading and the effect of individual variation on disease emergence. *Nature* **438**, 355–359 (2005).
3. Hogg, R. V., McKean, J. & Craig, A. T. *Introduction to mathematical statistics* (Pearson Education, 2005).
4. Sohail, M. S., Louie, R. H. Y., McKay, M. R. & Barton, J. P. MPL resolves genetic linkage in fitness inference from complex evolutionary histories. *Nature Biotechnology* **39**, 472–479 (2021).
5. Zhao, S. *et al.* Preliminary estimation of the basic reproduction number of novel coronavirus (2019-nCoV) in China, from 2019 to 2020: A data-driven analysis in the early phase of the outbreak. *International Journal of Infectious Diseases* **92**, 214–217 (2020).
6. Systrom, K., Vladek, T. & Krieger, M. Model powering rt.live. `https://github.com/rtcovidlive/covid-model` (2020).
7. Dietz, K. The estimation of the basic reproduction number for infectious diseases. *Statistical Methods in Medical Research* **2**, 23–41 (1993).
8. D'Arienzo, M. & Coniglio, A. Assessment of the SARS-CoV-2 basic reproduction number, R0, based on the early phase of COVID-19 outbreak in Italy. *Biosafety and Health* **2**, 57–59 (2020).
9. Delamater, P. L., Street, E. J., Leslie, T. F., Yang, Y. T. & Jacobsen, K. H. Complexity of the basic reproduction number (R0). *Emerging Infectious Diseases* **25**, 1–4 (2019).
10. Clark, S. J. & Perry, J. N. Estimation of the negative binomial parameter κ by maximum quasi-likelihood. *Biometrics* **45**, 309–316 (1989).
11. Saha, K. & Paul, S. Bias-corrected maximum likelihood estimator of the negative binomial dispersion parameter. *Biometrics* **61**, 179–185 (2005).
12. Hilbe, J. M. *Negative binomial regression* (Cambridge University Press, 2011).
13. Cui, Z. *et al.* Structural and functional characterizations of infectivity and immune evasion of SARS-CoV-2 Omicron. *Cell* **185**, 860–871.e13 (2022).
14. Qu, P. *et al.* Enhanced neutralization resistance of SARS-CoV-2 omicron subvariants bq. 1, bq. 1.1, BA.4.6, bf. 7, and BA.2.75. 2. *Cell host & microbe* **31**, 9–17 (2023).
15. Lin, X. *et al.* The NSP4 T492I mutation increases SARS-CoV-2 infectivity by altering non-structural protein cleavage. *Cell Host and Microbe* **31**, 1170–1184.e7 (2023).
16. Xia, H. *et al.* Evasion of type I interferon by SARS-CoV-2. *Cell Reports* **33**, 108234 (2020).
17. Hong, Q. *et al.* Molecular basis of receptor binding and antibody neutralization of Omicron. *Nature* **604**, 546–552 (2022).
18. Ramirez, S. *et al.* Overcoming culture restriction for SARS-CoV-2 in human cells facilitates the screening of compounds inhibiting viral replication. *Antimicrobial Agents and Chemotherapy* **65** (2021).
19. Qu, P. *et al.* Evasion of neutralizing antibody responses by the SARS-CoV-2 BA.2.75 variant. *Cell host & microbe* **30**, 1518–1526 (2022).
20. Saito, A. *et al.* Enhanced fusogenicity and pathogenicity of SARS-CoV-2 Delta P681R mutation. *Nature* **602**, 300–306 (2021).
21. Wang, Q. *et al.* Key mutations in the spike protein of SARS-CoV-2 affecting neutralization resistance and viral internalization. *Journal of Medical Virology* **95**, e28407 (2023).
22. Focosi, D., Spezia, P. G., Gueli, F. & Maggi, F. The era of the flips: How spike mutations l455f and f456l (and a475v) are shaping SARS-CoV-2 evolution. *Viruses* **16** (2024).
23. Escalera, A. *et al.* Mutations in SARS-CoV-2 variants of concern link to increased spike cleavage and virus transmission. *Cell Host and Microbe* **30**, 373–387.e7 (2022).
24. Syed, A. M. *et al.* Rapid assessment of SARS-CoV-2 evolved variants using virus-like particles. *Science* **374**, 1626–1632 (2021).
25. Iketani, S. *et al.* Antibody evasion properties of SARS-CoV-2 Omicron sublineages. *Nature* **604**, 553–556 (2022).
26. Liu, C. *et al.* Reduced neutralization of SARS-CoV-2 B.1.617 by vaccine and convalescent serum. *Cell* **184**, 4220–4236.e13 (2021).
27. McCallum, M. *et al.* N-terminal domain antigenic mapping reveals a site of vulnerability for SARS-CoV-2. *Cell* **184**, 2332–2347.e16 (2021).
28. Tamura, T. *et al.* Virological characteristics of the SARS-CoV-2 BA.2.86 variant. *Cell Host & Microbe* **32**, 170–180 (2024).
29. Sinha, S., Tam, B. & Wang, S. M. Rbd double mutations of SARS-CoV-2 strains increase transmissibility through enhanced interaction between rbd and ace2 receptor. *Viruses* **14**, 1 (2021).
30. Cheng, L. *et al.* Cross-neutralization of SARS-CoV-2 kappa and delta variants by inactivated vaccine-elicited serum and monoclonal antibodies. *Cell Discovery* **7**, 112 (2021).
31. Starr, T. N. *et al.* Shifting mutational constraints in the SARS-CoV-2 receptor-binding domain during viral evolution. *Science* **377**, 420–424 (2022). Publisher: American Association for the Advancement of Science.
32. Li, Q. *et al.* The impact of mutations in SARS-CoV-2 spike on viral infectivity and antigenicity. *Cell* **182**, 1284–1294 (2020).
33. Cao, Y. *et al.* BA.2.12.1, BA.4 and BA.5 escape antibodies elicited by Omicron infection. *Nature* (2022)(2022).
34. Deng, X. *et al.* Transmission, infectivity, and neutralization of a spike L452R SARS-CoV-2 variant. *Cell* **184**, 3426–3437.e8 (2021).
35. Suryadevara, N. *et al.* Neutralizing and protective human monoclonal antibodies recognizing the N-terminal domain of the SARS-CoV-2 spike protein. *Cell* **184**, 2316–2331.e15 (2021).
36. Alkhatib, M. *et al.* SARS-CoV-2 variants and their relevant mutational profiles: Update summer 2021. *Microbiology Spectrum* **9**, e01096–21 (2021).
37. Li, Y. *et al.* T-cell responses to SARS-CoV-2 omicron spike epitopes with mutations after the third booster dose of an inactivated vaccine. *Journal of Medical Virology* **94**, 3998–4004 (2022).
38. Greaney, A. J. *et al.* Mapping mutations to the SARS-CoV-2 RBD that escape binding by different classes of antibodies. *Nature Communications* **12**, 4196 (2021).
39. Tuekprakhon, A. *et al.* Antibody escape of SARS-CoV-2 Omicron BA.4 and BA.5 from vaccine and BA.1 serum. *Cell* **185**, 2422–2433.e13 (2022).
40. Ragonnet-Cronin, M. *et al.* Generation of SARS-CoV-2 escape mutations by monoclonal antibody therapy. *Nature Communications* **14**, 3334 (2023).
41. Wang, X. *et al.* 35B5 antibody potently neutralizes SARS-CoV-2 Omicron by disrupting the N-glycan switch via a conserved spike epitope. *Cell Host and Microbe* **30**, 887–895.e4 (2022).
42. Cerutti, G. *et al.* Cryo-EM structure of the SARS-CoV-2 Omicron spike. *Cell Reports* **38**, 110428 (2022).
43. Mohammad, A., Abubaker, J. & Al-Mulla, F. Structural modelling of SARS-CoV-2 Alpha variant (B.1.1.7) suggests enhanced furin binding and infectivity. *Virus Research* **303**, 198522 (2021).
44. Lista, M. J. *et al.* The P681H Mutation in the Spike Glycoprotein of the Alpha Variant of SARS-CoV-2 Escapes IFITM Restriction and Is Necessary for Type I Interferon Resistance. *Journal of Virology* **96**, e01250–22 (2022). Publisher: American Society for Microbiology.
45. de Silva, T. I. *et al.* The impact of viral mutations on recognition by SARS-CoV-2 specific t cells. *iScience* **24** (2021).
46. Elko, E. A. *et al.* Recurrent SARS-CoV-2 mutations at Spike D796 evade antibodies from pre-Omicron convalescent and vaccinated subjects. *Microbiology Spectrum* **12**, e03291–23 (2024). Publisher: American Society for Microbiology.
47. Haque, S. *et al.* Energetic and frustration analysis of SARS-CoV-2 nucleocapsid protein mutations. *Biotechnology and Genetic Engineering Reviews* 1–21 (2023).
48. Cao, Y. *et al.* Omicron escapes the majority of existing SARS-CoV-2 neutralizing antibodies. *Nature* **602**, 657–663 (2022).
49. Zhou, D. *et al.* The SARS-CoV-2 neutralizing antibody response to SD1 and its evasion by BA.2.86. *Nature Communications* **15**, 2734 (2024).
50. Cao, Y. *et al.* BA.2.12. 1, BA.4 and BA.5 escape antibodies elicited by omicron infection. *Nature* **608**, 593–602 (2022).

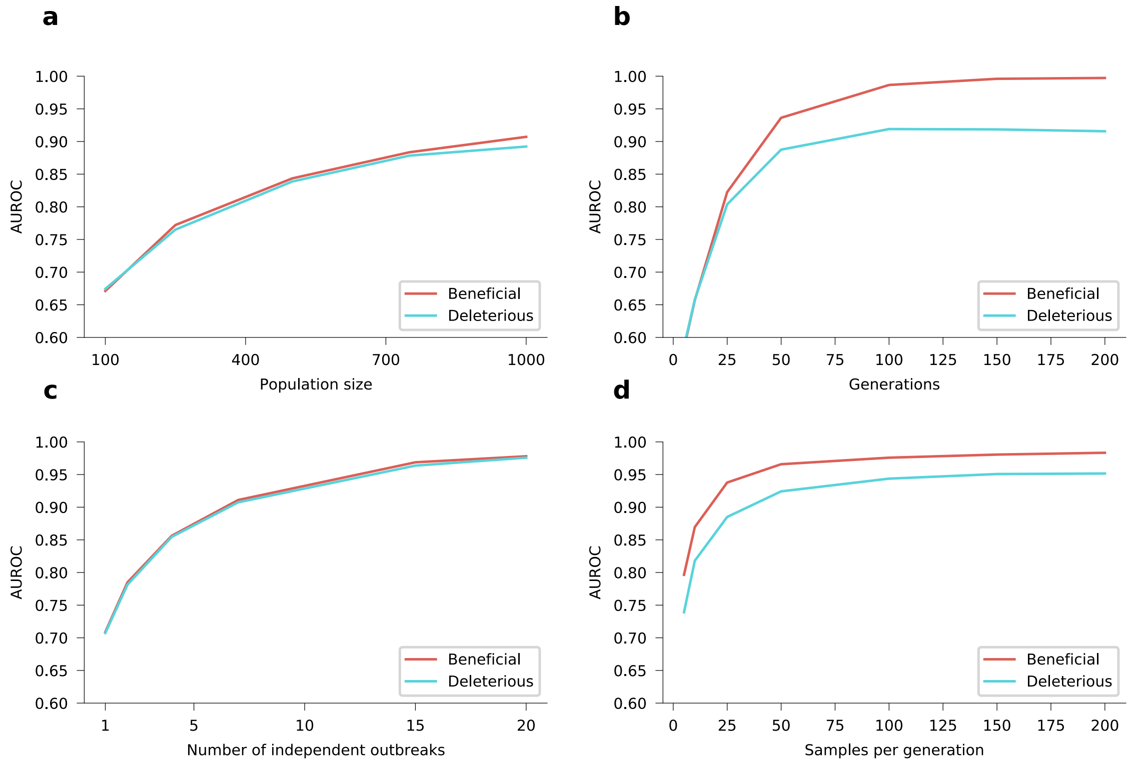

Supplementary Fig. 1. Accuracy of inference for different parameters. How the AUROC scores for both beneficial SNVs (in red) and deleterious SNVs (in blue) depends upon the different model parameters. **a**, Inference accuracy for different values of newly-infected population size. The parameters used are 10 simulations each with 50 sampled genomes per generation for 25 generations. **b**, Inference accuracy for different numbers of generations (serial intervals). Data is from a single simulation with 25 samples per generation and a newly-infected population size of 10,000. **c**, Inference accuracy for different numbers of independent outbreaks (simulations). The parameters used are 50 samples per generation for 10 generations and a newly-infected population size of 10,000. **d**, Inference accuracy for different values of samples per generation. Data is from a single simulation with 50 generations with a newly-infected population size of 10,000. The initial population is a mixture of two variants with beneficial SNVs ($s = 0.03$), two with neutral SNVs ($s = 0$), and two with deleterious SNVs ($s = -0.03$). Dispersion parameter k is fixed at 0.1. This is the same initial population composition as described in **Fig. 1**. All AUROC scores are calculated by averaging over 1,000 replicate simulations.

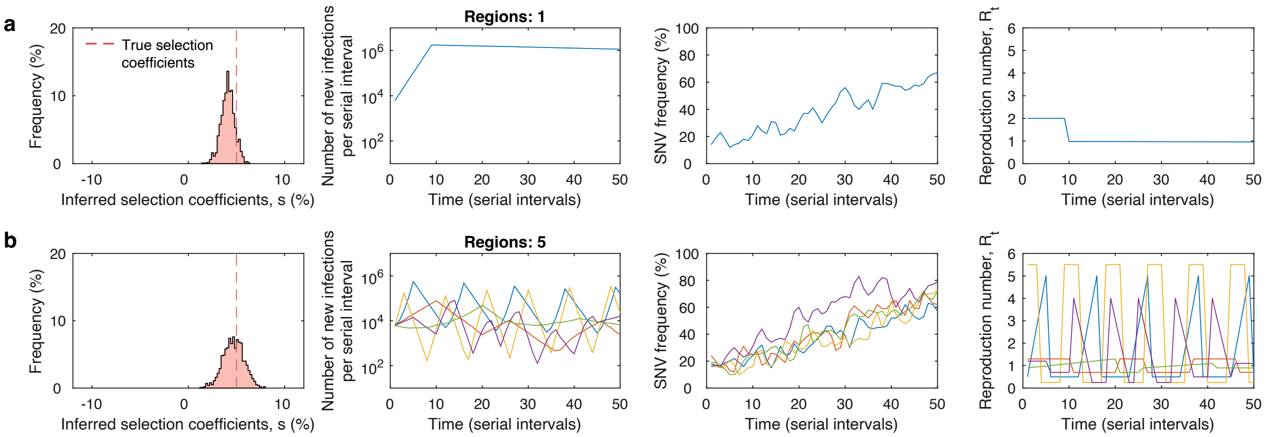

Supplementary Fig. 2. Inference is robust to variation of reproduction number, R, across regions. Our approach provides a systematic way to combine data from outbreaks in multiple regions. Simulations show that the estimator (15) in **Methods** has good performance whether the selection coefficients are inferred based on data from, **a**, a single region or, **b**, five regions. *Simulation parameters.* The initial population in each region is a mixture of a neutral variant with no mutations and a variant with a beneficial SNV ($s = 0.05$). The same beneficial SNV appears in all 5 regions. Each region has a different profile of the time-varying reproduction number, R (rightmost panel). In the first simulation, the number of newly infected individuals per serial interval rises rapidly from 6,000 to around 10,000 and stays nearly constant thereafter. While in the second simulation it has a different profile for each region, all the while staying between 100 and 100,000. Dispersion parameter k is fixed at 0.1 for both simulation scenarios.

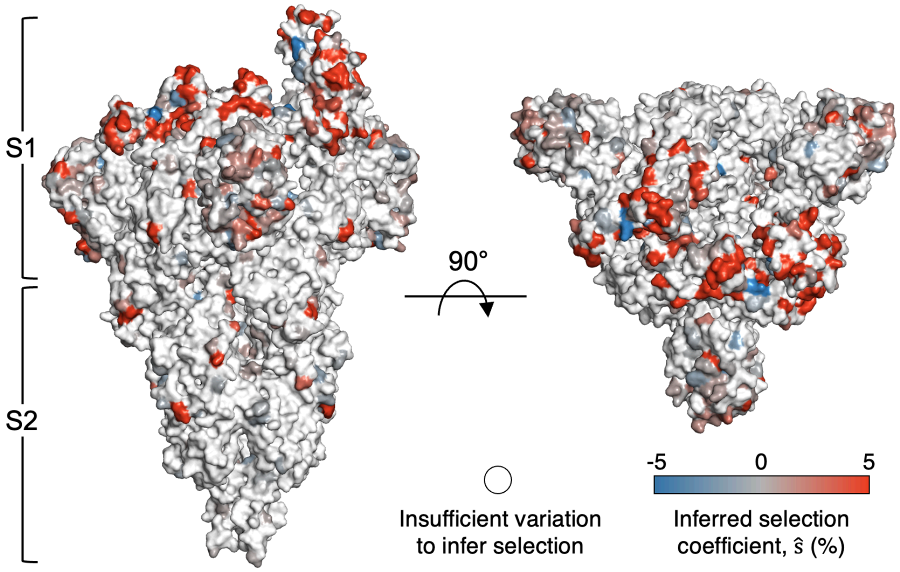

Supplementary Fig. 3. Inferred selection coefficients for Spike mutations mapped on the crystal structure. The majority of the inferred strongly selected mutations are in the S1 subunit of Spike. For sites with multiple mutations, the mutation with the largest magnitude of inferred selection coefficient was used for mapping. Structure of the Spike protein was obtained from `http://rcsb.org/` (PDB ID: 7WG7) (ref. [13]).

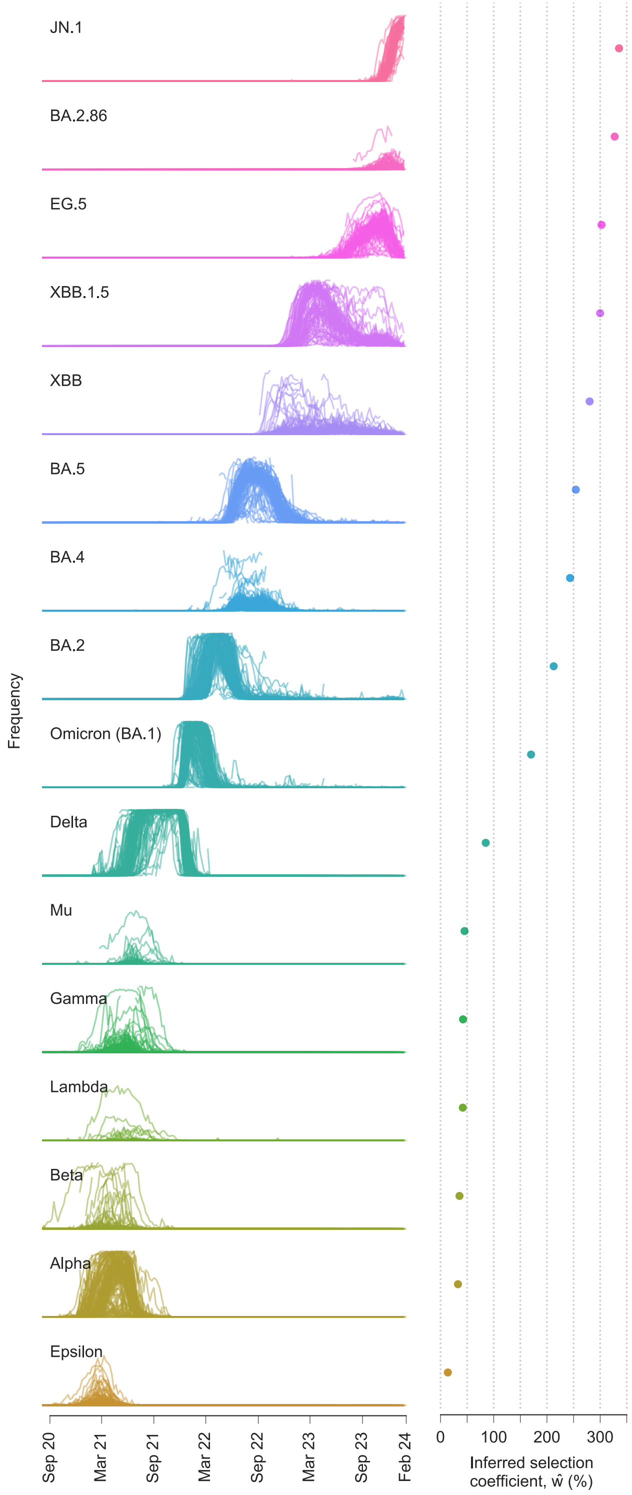

Supplementary Fig. 4. Multiple SARS-CoV-2 variants strongly increase transmission rate. Frequencies of major variants and their total inferred selection coefficients, shown as mean values ± one s.d. from bootstrap subsampling of regional data (**Methods**), defined relative to the WIV04 reference sequence. Selection coefficients for variants with multiple SNVs are obtained by summing the effects of all variant-defining SNVs. Because our method uses global data and accounts for competition between variants, we infer large transmission advantages even for variants such as Gamma, Beta, Lambda, and Epsilon, which never achieved the same level of global dominance as variants such as Alpha and Delta.

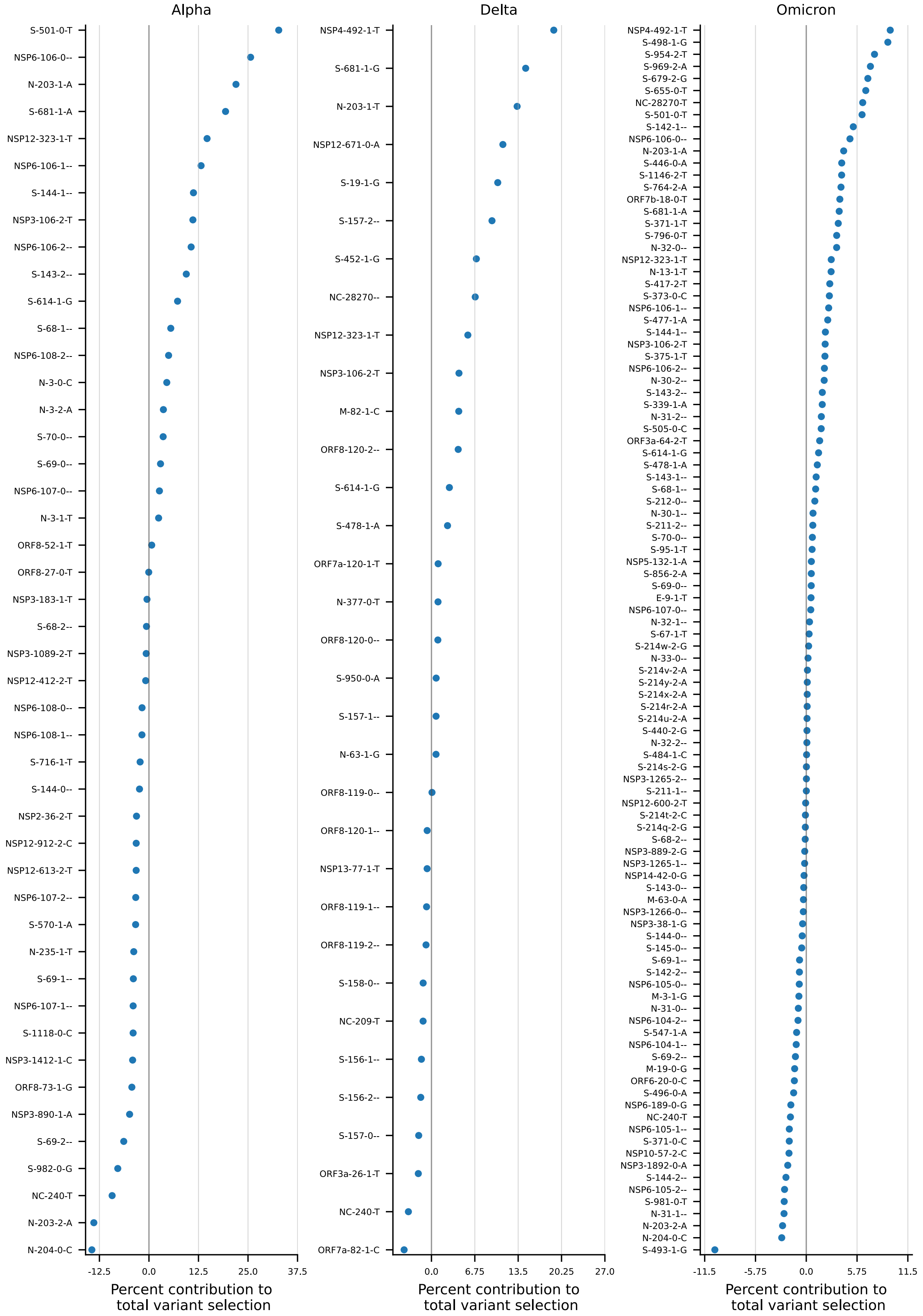

Supplementary Fig. 5. For major variants, a minority of SNVs provide most of the total increase in transmission. Fraction of the total increase in transmission for Alpha, Delta, and Omicron (BA.1) provided by each variant-defining mutation. For each variant, a few strongly beneficial mutations provide most of the total increase in transmission. Most other mutations are inferred to be nearly neutral. For some variants, a small number of mutations are inferred to be substantially deleterious.

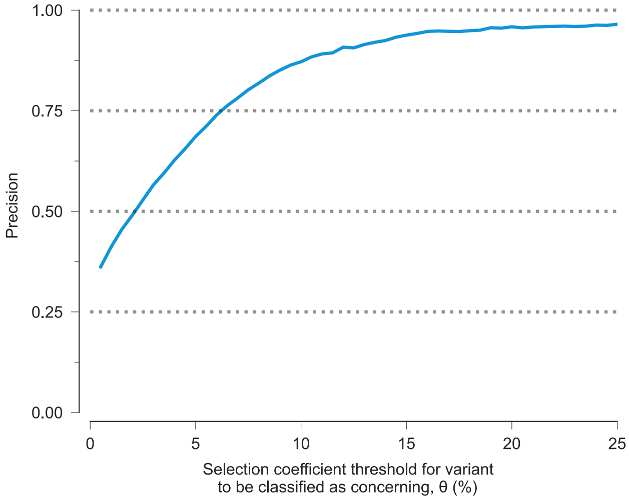

Supplementary Fig. 6. Variants with large inferred selection coefficients are overwhelmingly likely to belong to major variants, even when selection is estimated as data becomes available. Fraction of variants classified as concerning with SNVs that belong to major SARS-CoV-2 variants, plotted as a function of the selection coefficient threshold θ used for classification. We consider (groups of) SNVs classified as concerning to be true positives if they belong to major variants and false positives otherwise. With this definition, this fraction is equivalent to the precision for classification.

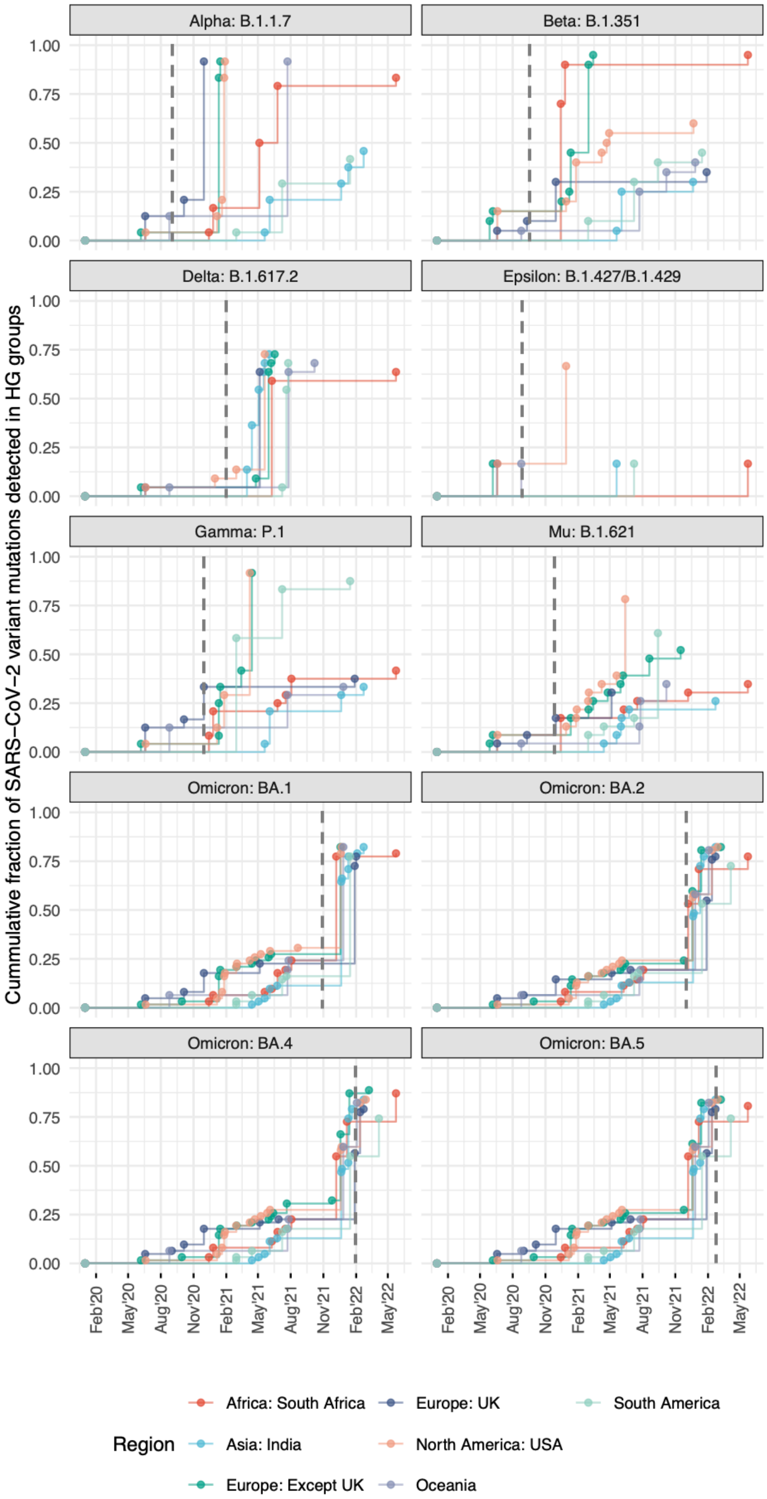

Supplementary Fig. 7. Cumulative fraction of SARS-CoV-2 variant-defining mutations identified as HG across regions. Results are shown for 10 major variants across 7 broad geographical regions. The vertical dashed line indicates the earliest sample date for each variant. Data of variant-defining mutations and their earliest sample dates were obtained from https://covariants.org.

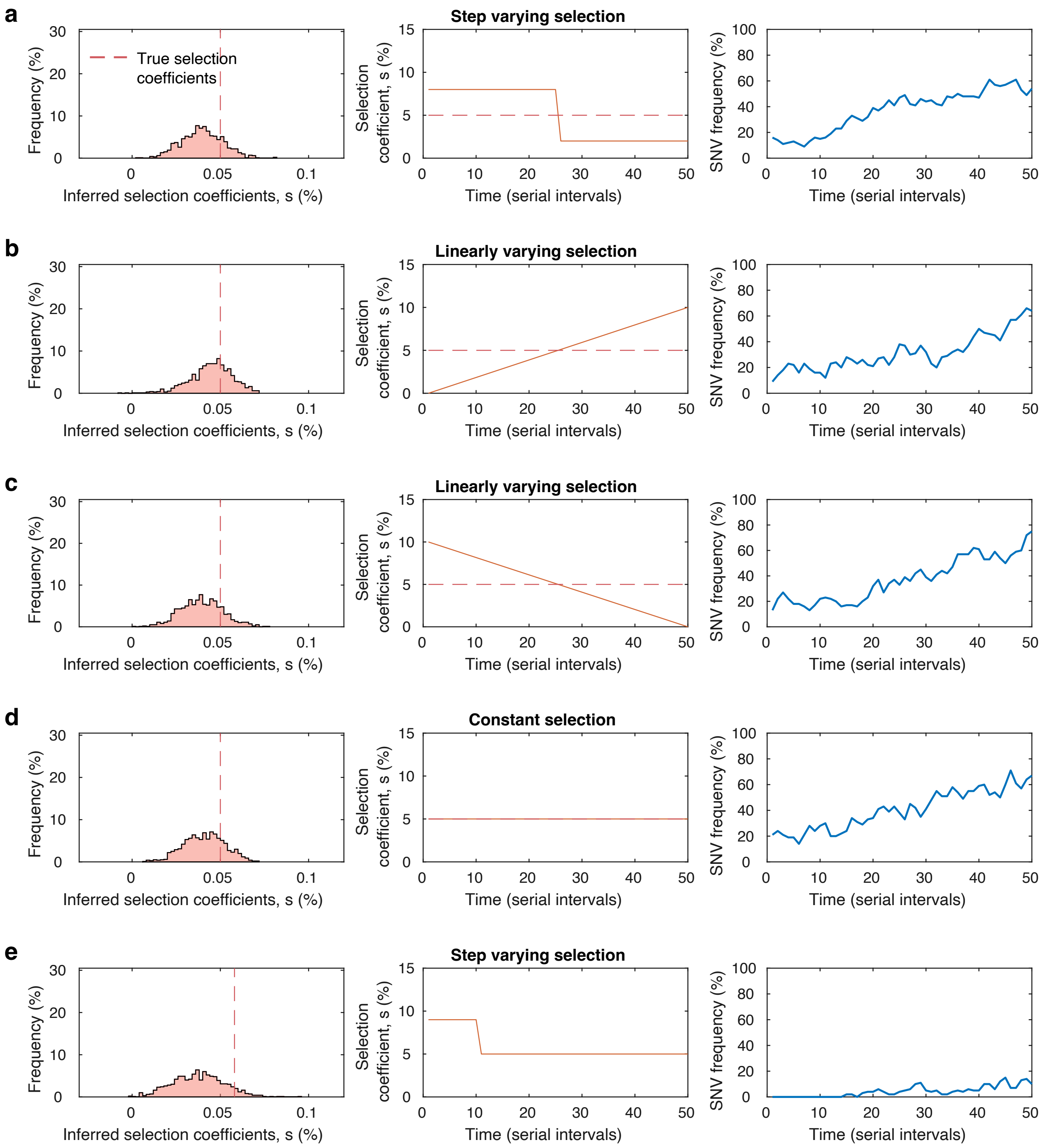

Supplementary Fig. 8. Average value inferred for time-varying selection coefficients. We simulated five scenarios of time-varying selection coefficients: **a**, step varying, **b**, linearly increasing, **c**, linearly decreasing, **d**, constant over time and, **e**, step varying where the SNV appears in the population after the true selection coefficient has changed. In each case, the inferred selection coefficient is close to the average of the time-varying selection coefficient over the time when the SNV was present in the population. *Simulation parameters*. The initial population in the first four simulation scenarios is a mixture of a neutral variant with no mutations and a variant with a beneficial SNV with a time-varying selection coefficient (center panels). In the fifth simulation scenario, the initial population consists entirely of the neutral variant with the beneficial mutant appearing after 15 serial intervals. The number of newly infected individuals per serial interval rises rapidly from 6,000 to around 10,000 and stays nearly constant thereafter. Dispersion parameter k is fixed at 0.1 for all simulation scenarios.

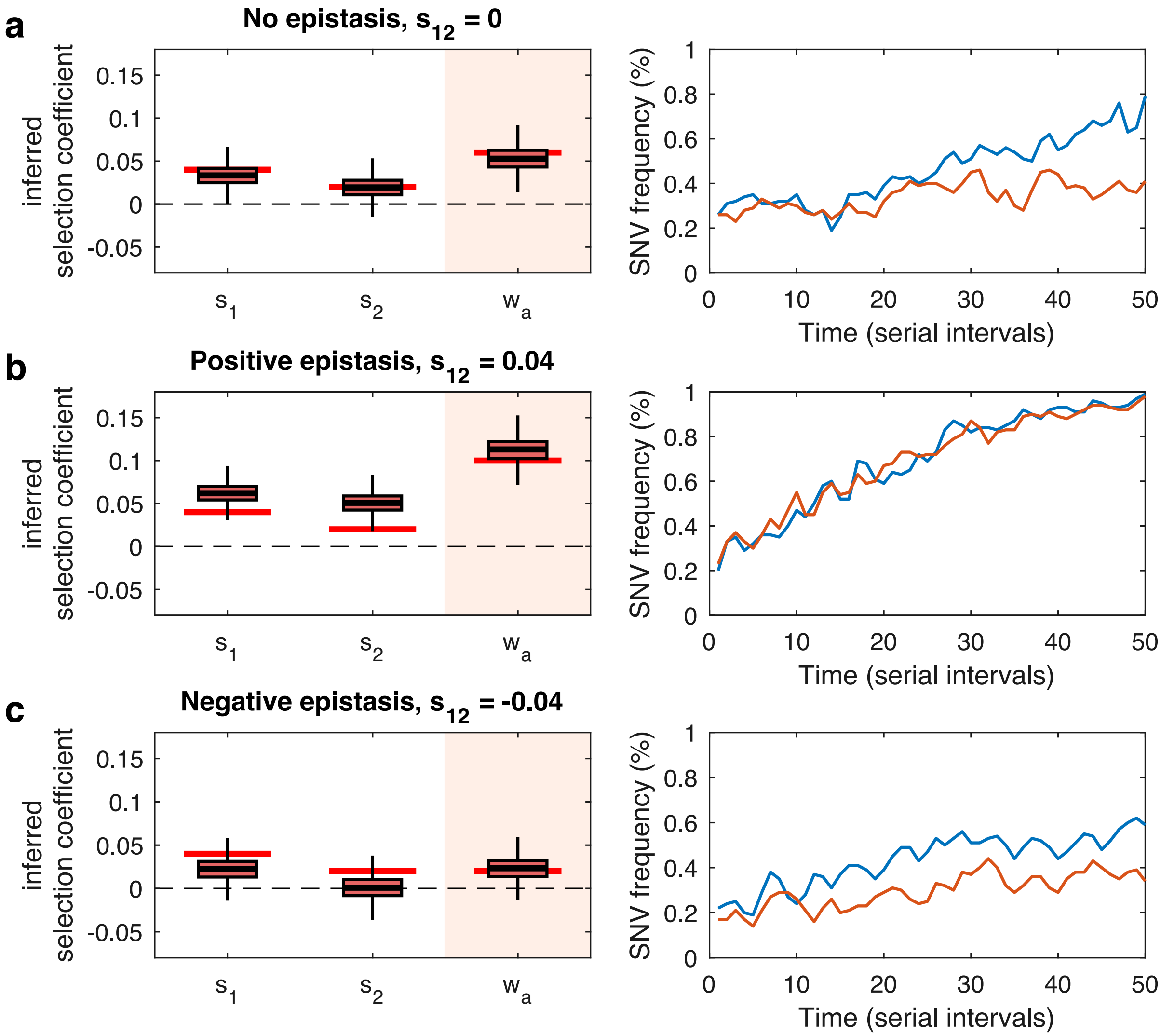

Supplementary Fig. 9. Accurate inference of variant fitness in the presence of epistasis. a, Both SNV selection coefficients and variant selection coefficients are inferred accurately in the absence of epistasis. Inferred selection coefficients over 1,000 runs are shown in box plots, with true values for the parameters shown with solid bars in red. The lower and upper edge of the box plot correspond to the 25th to 75th percentiles, the bar inside the box plot corresponds to the median while the top and bottom whiskers show the maximum and minimum value within 1.5 times the interquartile range. In scenarios with positive epistasis (**b**) or negative epistasis (**c**), our method attributes the effect of epistasis to selection coefficients. Thus, while the inferred SNV selection coefficients may be under- or over-estimated, the inferred variant selection coefficients are recovered. *Simulation parameters*. We simulate a two-locus system where the initial population consists of a mixture of all four variants, i.e., a neutral variant with no mutations, a variant with two beneficial SNVs ($s_1 = 0.04$, $s_2 = 0.02$), and both single SNV variants. The initial frequencies in the population of the neutral, the two single mutant variants, and the double mutant variants are set to 67%, 10%, 10%, and 13%. We simulate three scenarios with the epistasis term taking on values s_{12} = {0, 0.04, -0.04}. Here the selection coefficient for the double mutant is $s_1 + s_2 + s_{12}$. The number of newly infected individuals per serial interval rises rapidly from 6,000 to around 10,000 and stays nearly constant thereafter. Dispersion parameter k is fixed at 0.1 for all simulation scenarios.

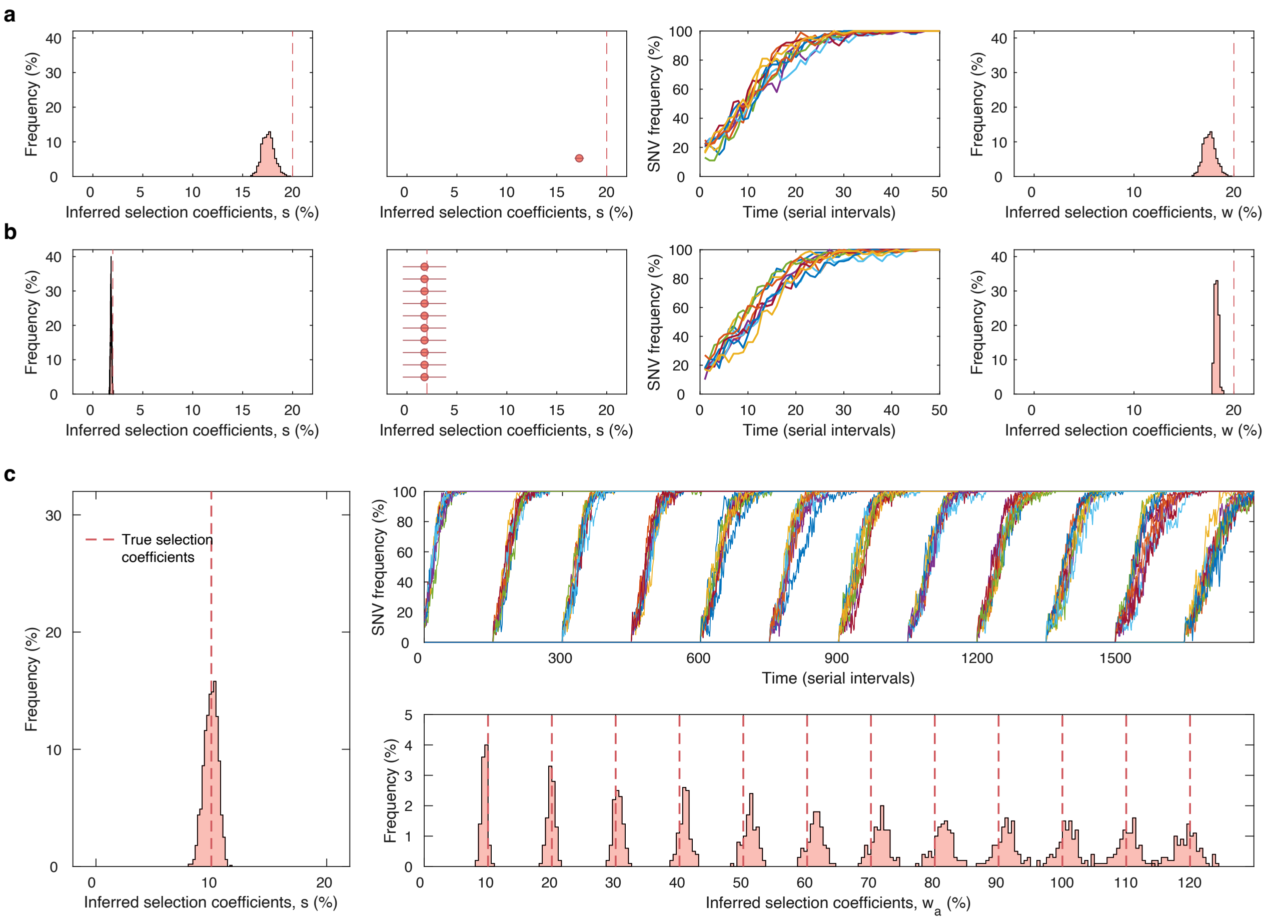

Supplementary Fig. 10. Ability to estimate large variant selection coefficients, w_a. While the estimate (15) in **Methods** is derived assuming selection coefficients are small, simulations show that combining data from multiple regions allows for accurate estimation of both large SNV selection coefficients, s, and variant selection coefficients, w_a. **a**, A scenario with a variant containing a single strongly beneficial SNV ($s = 0.2$) and, **b**, a scenario with a variant containing 10 mildly beneficial SNVs ($s = 0.02$). The true variant selection coefficient w_a has the same magnitude in both simulation scenarios ($w_a = 0.2$). **c**, Simulating a scenario where 12 beneficial SNVs ($s = 0.1$) appear and fixate successively (top right panel), such that w_a ranges from 0.1 to 1.2, both the SNV (left panel) and variant selection coefficient (bottom right panel) were estimated accurately. Results are obtained by combining data from 10 regions. Histograms are obtained from $1,000$ replicate simulations. *Simulation parameters.* In the simulation scenarios considered in **a** and **b**, the initial population in each region consists of a mixture of a neutral variant with no mutations along with a variant with a single strongly beneficial SNV (s = 0.2), or a variant with 10 beneficial SNVs (s = 0.02) respectively. In the simulation in **c**, each region's initial population consists of a mixture of a neutral variant with no mutations along with a variant with beneficial mutations. In this latter variant, 12 beneficial mutations (s = 0.1) appear and fixate in succession such that the variant selection coefficient varies from w_a = 0.1 to w_a = 1.2. The same variant appears in 10 independent regions in all simulation scenarios. The number of newly infected individuals per serial interval is nearly constant around 10,000. Dispersion parameter k is fixed at 0.1.

Number of new infections per serial interval	Sampling	Inference Parameter (n)	AUROC Beneficial	AUROC Deleterious
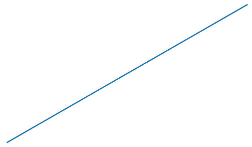	Finite	Time-Varying	0.832	0.779
		**Constant**	**0.937**	**0.881**
	Perfect	**Time-Varying**	**0.999**	**0.992**
		Constant	0.973	0.940
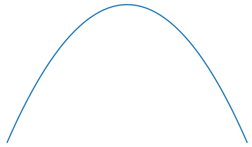	Finite	Time-Varying	0.873	0.821
		**Constant**	**0.944**	**0.882**
	Perfect	**Time-Varying**	**1.0**	**0.999**
		Constant	0.986	0.950
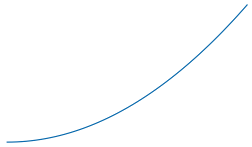	Finite	Time-Varying	0.798	0.736
		**Constant**	**0.873**	**0.824**
	Perfect	**Time-Varying**	**0.981**	**0.935**
		Constant	0.905	0.863

Supplementary Fig. 11. Effects of finite sampling on inference using constant and time-varying parameters. The ability of the model to distinguish beneficial and deleterious SNVs, as measured by the AUROC score, depending on whether the sampling is perfect or finite and whether constant parameters or the true time-varying parameters are used for the number of new infections per serial interval n in the inference. If parameters are considered to be constant, then these parameters are not required for inference using (15) in **Methods**. Both simulations use constant values of $k = 0.01$ and $R = 1$. The results are similar but less dramatic if the correct time-varying values are used for k or R as well. Results are shown for different trajectories of numbers of infections and are consistent regardless of the trajectory. In the upper panel, the number of new infections per serial interval, n, starts at $5,000$ and rises linearly to $100,000$. In the middle panel, n starts at $10,000$, rises quadratically to a maximum of $200,000$, and then falls back to the original number. In the final panel, n rises from an initial size of $1,000$ to a final size of $65,000$. All simulations are run for 50 serial intervals. Rows that yield better inference are marked by bold text. If sampling is finite, then it is better to use constant parameters; if sampling is perfect, then it is better to use the real time-varying parameters. The initial population of individuals are infected with a mixture of two variants with beneficial SNVs ($s = 0.03$), two with neutral SNVs ($s = 0$), and two with deleterious SNVs ($s = -0.03$), as in **Fig. 1**. Simulations are run for 50 simulations with 25 samples in each serial interval, and AUROC scores are averaged over 1,000 replicate simulations.

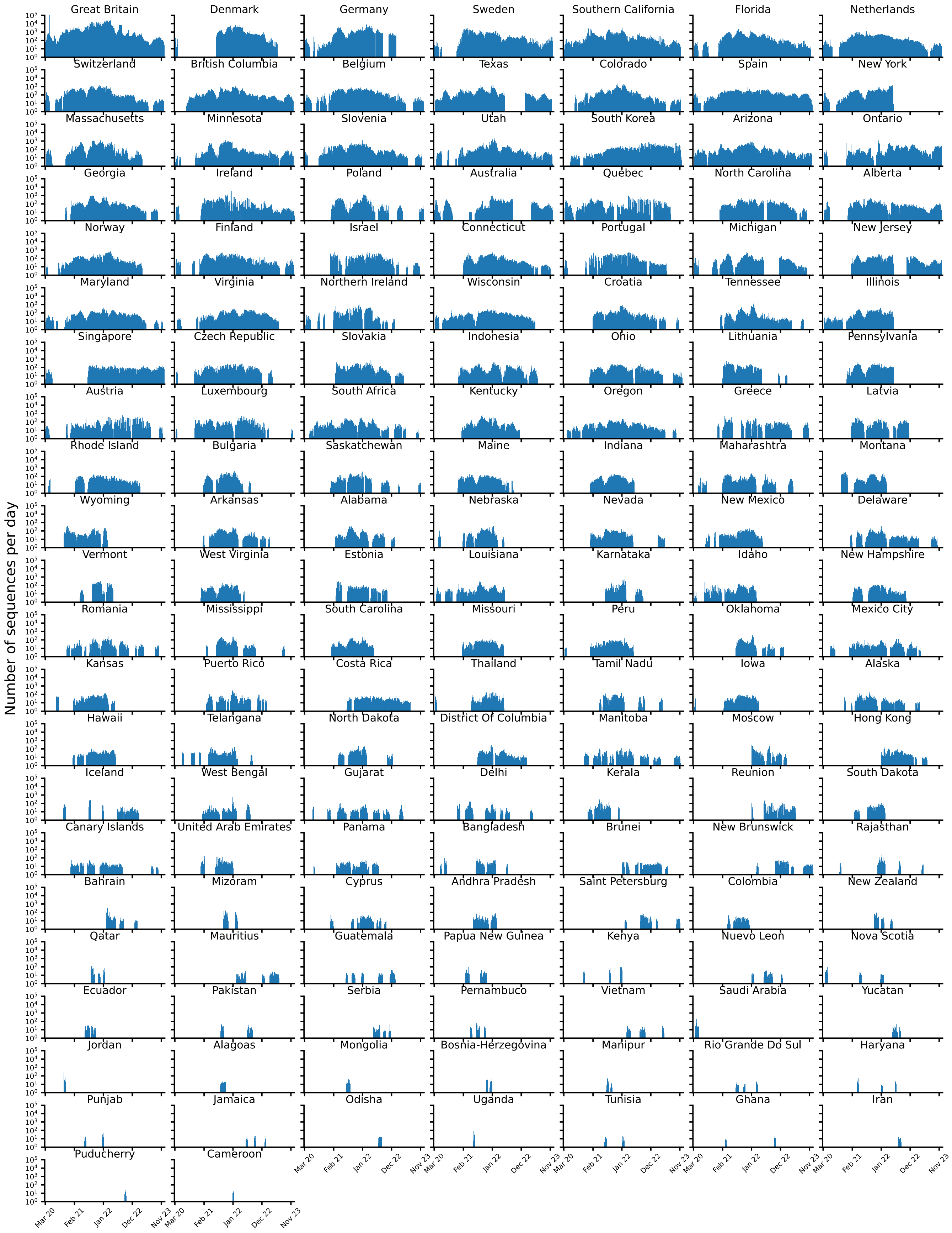

Supplementary Fig. 12. Sampling Distributions. The number of genomes per day in the regions that are used for inference.

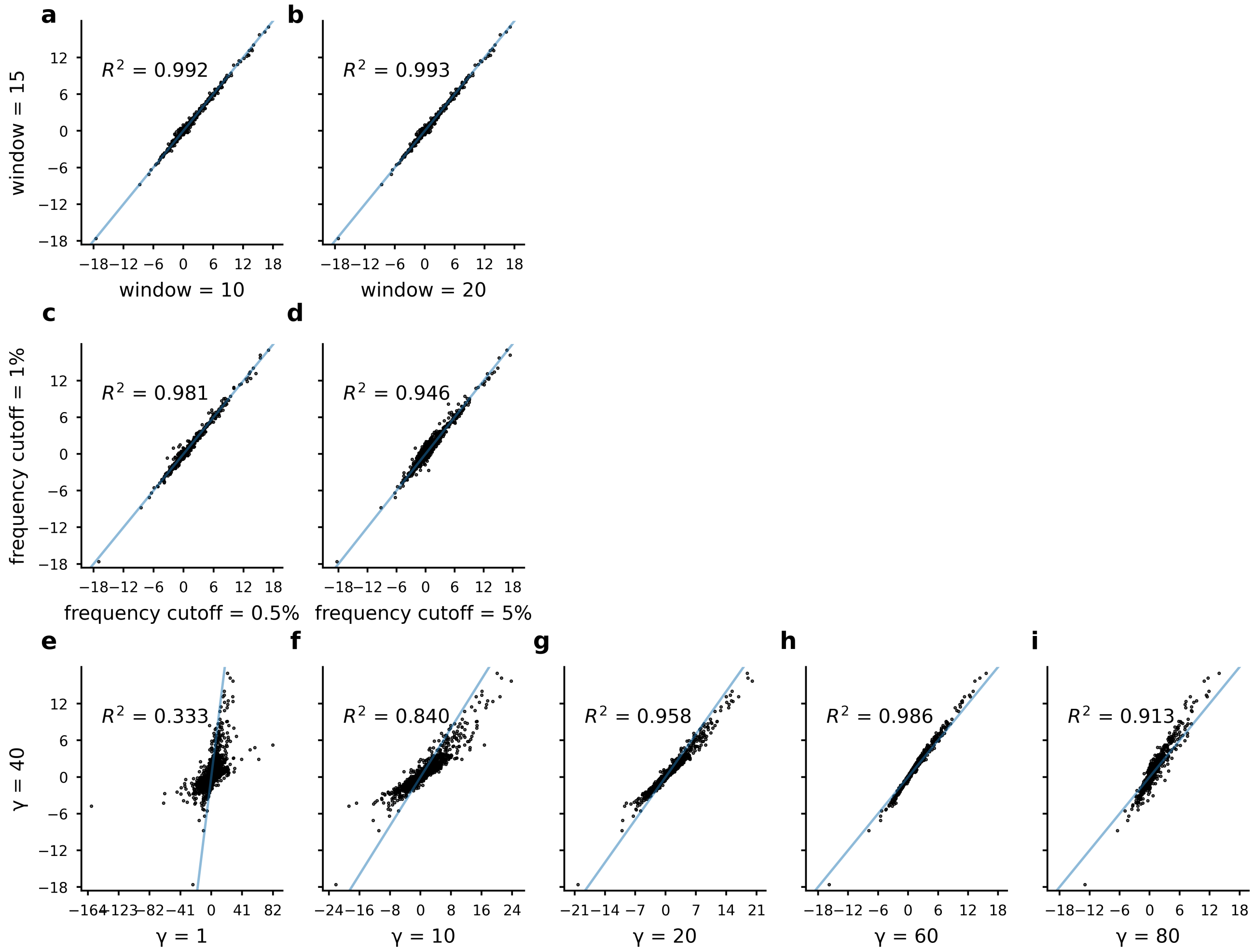

Supplementary Fig. 13. Inferred selection coefficients are robust to different values of the regularization γ', different frequency cutoffs, and different numbers of days used to calculate the frequency changes. a-b, Comparison of inferred coefficients when the number of days at the beginning and end of the time-series are used in order to calculate the frequency changes. Inferred coefficients are largely robust to these changes **c-d**, Comparison of inferred coefficients for different frequency cutoffs. Including more or less sites does not alter the order of inferred coefficients. **e-i**, Comparison of inferred coefficients for different values of the regularization. Altering the regularization value has little effect upon the distribution of inferred selection coefficients, and selection coefficients for different values of the regularization are highly correlated.

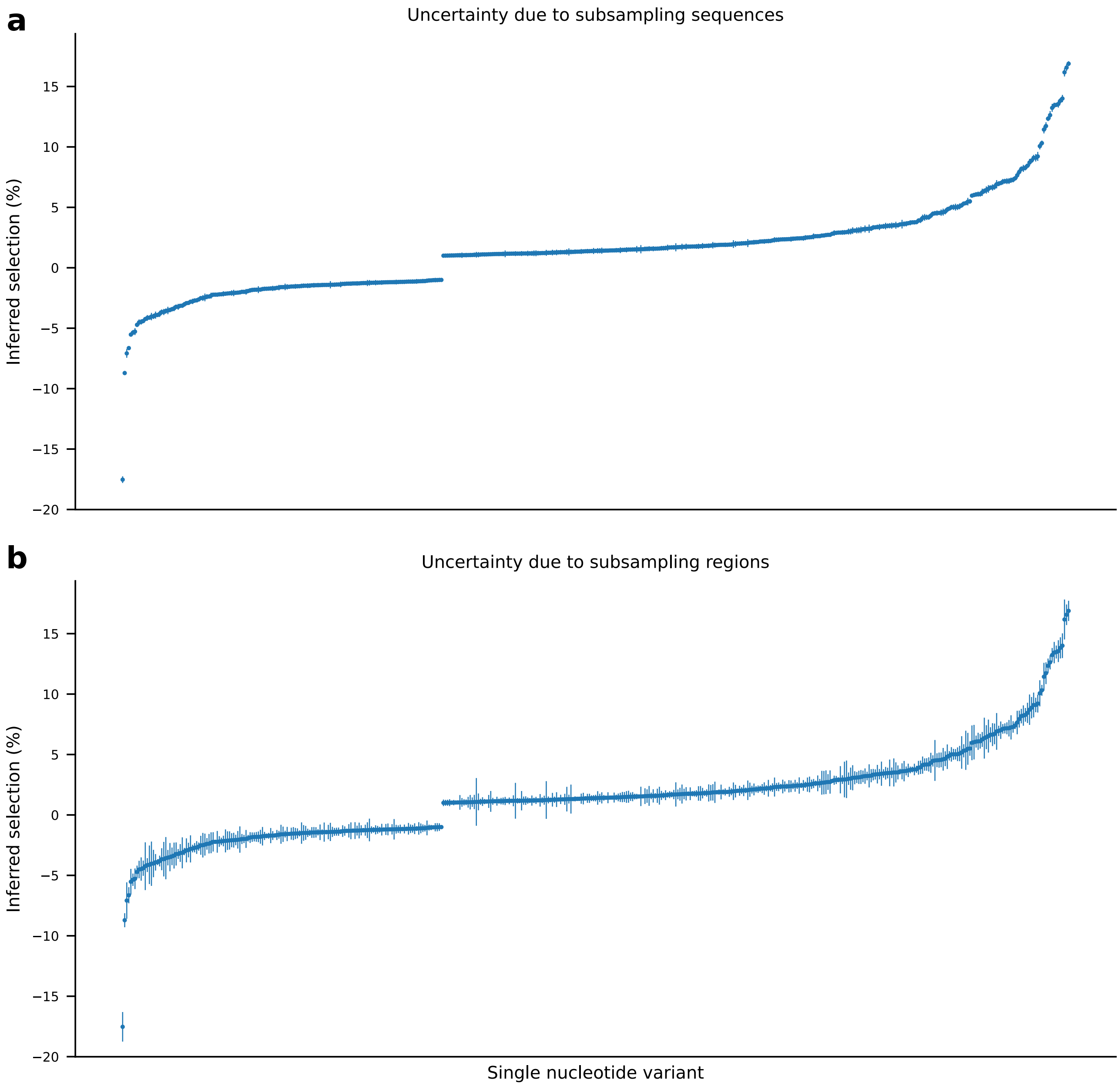

Supplementary Fig. 14. Selection coefficient estimates and uncertainty. Plots of all inferred selection coefficients with absolute values greater than 1%. **a**, Selection coefficients with uncertainty estimates from bootstrapping the sequences in each region. 20 sequences were sampled per time point per region, with replacement. Error bars represent standard deviations of the inferred coefficients computed over 100 bootstrap samples. **b**, Selection coefficients with uncertainty estimates from subsampling the regions used. For each run, we inferred selection coefficients using a random subsample of 80% of the total number of regions. Error bars represent standard deviations of the inferred coefficients computed over 100 samples.

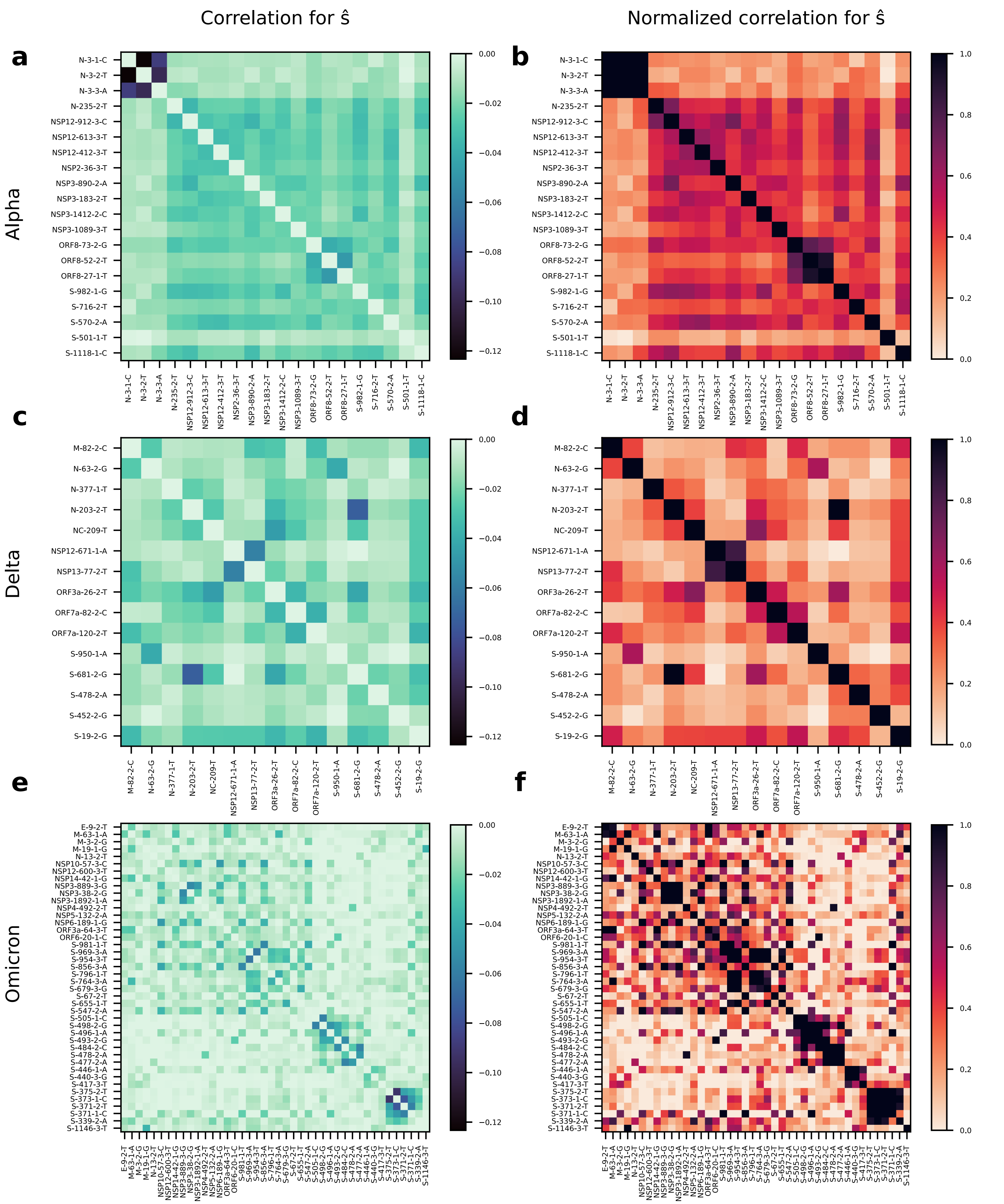

Supplementary Fig. 15. Example correlations between $\hat{s}$ for strongly linked subsets of mutations defining major variants. As discussed in **Supplementary Information**, the covariance of the inferred parameters is given by the matrix in (5). The correlation matrix of the inferred parameters is easily calculated from this covariance. SNV labels are in the format of xxx-yyy-z-n, where xxx is the protein, yyy is the codon in the protein, z is the index of the nucleotide in the codon, and n is the nucleotide. **a, c, e**, The correlation matrix for SNVs that are strongly linked to one another in Alpha, Delta, and Omicron, respectively. The diagonal elements, all equal to 1 in a correlation matrix, are set to zero for visualization purposes. **b, d, f**, Correlation matrices from **a**, **c**, and **e**, normalized by the maximum possible correlation for a group of linked SNVs, as discussed in **Supplementary Information**, with the same number of SNVs. The (i, j)th element of these matrices represents the percent of linkage between the selection coefficients for SNVs i and j.

Rank	Protein	SNV(s) (nt)	Mutation (aa)	Selection (%)	Location	Associated variant(s)	Phenotypic effect
1	S	T23018C/ T23019C	F486P	16.9 ± 0.8	RBM	XBB.1.5, EG.5.1, BA.2.86, JN.1	Reduces recognition by neutralizing antibodies [14]
2	NSP4	C10029T	T492I	16.6 ± 0.9		Delta, Lambda, Mu, BA.1 (and subvariants)	Increased viral replication capacity and infectivity, cleavage efficiency of the viral protease, and antibody evasion [15]
3	NSP6	Δ 11288-90	Δ106	16.5 ± 0.8		Alpha, Beta, Gamma, Eta, Iota, Lambda, BA.1 (and subvariants)	*Increased transmission by interferon antagonism [16]
4	S	A23055G	Q498R	16.2 ± 1.7	RBM	BA.1 (and subvariants)	Increased ACE2 binding and resistance to nAbs [13,17]
5	S	A24424T	Q954H	14.0 ± 1.0	HR1	BA.1 (and subvariants)	Increased infectivity in vitro [18]
6	S	T22942A	N460K	13.8 ± 0.9	RBM	XBB, XBB.1.5, EG.5.1, HK.3, BA.2.86, JN.1	Enhanced neutralization resistance, enhanced spike processing and cell-cell fusion, improves ACE2 binding [19]
7	S	C23604G	P681R	13.6 ± 0.9	FCS	Delta, Kappa, BA.2.86, JN.1	Enhanced cleavage, fusogenicity, and pathogenicity [20]
8	S	G22599C	R346T	13.5 ± 0.5	RBD	XBB, XBB.1.5, EG.5.1, HK.3	Evasion of antibody recognition [14]
9	S	T24469A	N969K	13.2 ± 0.6	HR1	BA.1 (and subvariants)	Improved structural stability [13]
10	S	T23599A	N679K	12.6 ± 0.6	FCS	BA.1 (and subvariants)	*Increased proteolytic activation [13]
11	S	G22927C	L455F	12.3 ± 0.4	RBM	HK.3 (L455S in JN.1)	Enhanced resistance to immune sera [21], increased ACE2 binding [22]
12	S	C23525T	H655Y	11.7 ± 0.9	FCS	Gamma, BA.1 (and subvariants)	Increased viral replication, spike protein cleavage, and transmission in vivo [23]
13	N	G28881T	R203M	11.4 ± 1.2		Delta, Kappa	Enhanced replication, RNA delivery and packaging [24]
14	S	G22599A	R346K	10.3 ± 0.5	RBD	Mu, BA.1, XBB, XBB.1.5, EG.5.1, HK.3	Reduced neutralization [25]
15	S	A23063T	N501Y	10.1 ± 1.1	RBM	Alpha, Beta, Gamma, Mu, BA.1 (and subvariants)	Increased infection, transmission, ACE2 binding, and resistance to nAbs [13]
16	S	C21618G	T19R	9.2 ± 0.7	NTD	Delta	*Increased resistance to NTD-specific nAbs [26,27]
17	NSP12	G15451A	G671S	9.1 ± 0.6		Delta, XBB, XBB.1.5, EG.5.1, HK.3	
18	S	T22928C	F456L	8.9 ± 0.9	RBM	HK.3	Enhanced resistance to immune sera [21], increased ACE2 binding [22]
19	S	C21618T	T19I	8.7 ± 1.3	NTD	BA.2 (and subvariants)	*Increased resistance to NTD-specific nAbs [26,27]
20	S	A22910G	N450D	8.3 ± 0.6	RBM	BA.2.86, JN.1	Increased ACE2 binding [28]
21	S	C22995G	T478K	8.2 ± 0.8	RBM	BA.1 (and subvariants)	T478K enhances ACE2 binding [29], and enhances neutralization resistance [30]
22	S	C22916A	L452M	8.2 ± 0.6	RBM	BA.2 subvariants; L452W in BA.2.86 and JN.1, L452R in BA.4, BA.5	Increased RBD expression (stability) [31], increased resistance to nAbs [32,33], and increased cell entry [34]
23	NSP6	T11296G	F108L	7.6 ± 1.0			*Increased transmission by interferon antagonism [16]
24	S	Δ21986-88	Δ142	7.6 ± 1.0	NTD	BA.1 (G142D in BA.2 and subvariants)	*Increased resistance to NTD-specific nAbs [35]
25	S	C22033A	F157L	7.4 ± 0.3	NTD	BA.2.75 (F157S in BA.2.86 and JN.1)	In epitope recognized by neutralizing antibodies [36]
26	S	A22893G	K444R	7.3 ± 0.5	RBM		Increased resistance to immune sera [21], evasion of antibody recognition [14]
27	N	G28881A	R203K	7.2 ± 1.0		Alpha, Gamma, Lambda, BA.1 (and subvariants)	Enhanced replication, RNA delivery and packaging [24]
28	S	Δ 22031	Δ157	7.2 ± 0.5	NTD	Delta	In epitope recognized by neutralizing antibodies [36]
29	S	G22577C	G339H	7.2 ± 0.7	RBD	XBB.1.5, BA.2.75, EG.5.1, HK.3, BA.2.86, JN.1	G339D Interferes with T-cell response [37]
30	S	T23018G	F486V	7.2 ± 0.5	RBM	BA.4, BA.5	Increased ACE2 binding and resistance to nAbs [38,39]
31	S	T22917A	L452Q	7.1 ± 0.4	RBM	Lambda, BA.2.12.1	Increased RBD expression (stability) [31], increased resistance to nAbs [32,33], and increased cell entry [34]
32	S	T22896A	V445H	7.0 ± 0.7	RBM	BA.2.86, JN.1	Enhanced resistance to immune sera [21], increased ACE2 binding [22]
33	S	A22629C	K356T	7.0 ± 0.4	RBD	BA.2.86, JN.1	Neutralization of immune sera [40]
34	S	C23854A	N764K	6.9 ± 1.5		BA.1 (and subvariants)	Improved structural stability [41,42]
35	N	Δ28367-69	Δ32	6.9 ± 1.4		BA.1 (and subvariants)	
36	S	C23604A	P681H	6.7 ± 0.9	FCS	Alpha, Mu, BA.1 (and subvariants, BA.2.66 and JN.1 have P681R)	Enhanced cleavage [43] and increased resistance to interferon-induced immunity [44], leading to increased replication and/or transmission
37	S	G22898A	G446S	6.6 ± 0.9	RBM	XBB, XBB.1.5, EG.5.1, HK.3, BA.2.86, JN.1	enhanced resistance to neutralizing antibodies [19]
38	NSP6	T11288A	S106T	6.6 ± 0.6			*Increased transmission by interferon antagonism [16]
39	N	C28311T	P13L	6.5 ± 1.4		Lambda, BA.1 (and subvariants)	Escape from a HLA-B*27:05 $CD8^+$ T cell epitope [45]
40	S	G23948T	D796Y	6.4 ± 1.0		BA.1 (and subvariants)	Improved structural stability [13] and antibody evasion [46]
41	N	C28367T	R32C	6.4 ± 1.7			Alters frustration state of virus and may affect stability, function, and pathogenicity [47]
42	S	C22674T	S371F	6.2 ± 0.6	RBD	BA.2 (and subvariants)	Increased resistance to nAbs [33,48]
43	S	T22917G	L452R	6.1 ± 0.6	RBM	Delta, Kappa, Epsilon, BA.4, BA.5	Increased RBD expression (stability) [31], increased resistance to nAbs [32], and increased cell entry [33]
44	S	A22893C	K444T	6.1 ± 0.7	RBM	BQ.1	Increased resistance to immune sera [21], evasion of antibody recognition [14]
45	S	G23222A	E554K	6.1 ± 0.5	RBM	BA.2.86, JN.1	Escape from monoclonal antibodies [49]
46	S	C22295A	H245N	6.0 ± 1.4	NTD	BA.2.86, JN.1	Significantly increases ACE2 binding [28]
47	S	G22775A	D405N	5.5 ± 1.3	RBD	BA.2 (and subvariants)	Escapes many neutralizing antibodies [50]
48	S	C22353A	A264D	5.3 ± 1.6	NTD	BA.2.86, JN.1	Increases ACE2 binding [28]
49	S	G21987A	G142D	5.3 ± 0.5	NTD	BA.2 (and subvariants), BA.4, BA.5	Increased resistance to NTD-specific nAbs [13,35]
50	S	G24368T	D936Y	5.2 ± 1.3			Increased infectivity [32]

Supplementary Table 1. Highly selected amino acid substitutions across the SARS-CoV-2 genome. Error are standard deviations of inferred selection coefficients across 100 replicate sub-samples of 80% of the original regions. * represents cases where the phenotypic effect of an amino acid variant has not been reported explicitly in the literature. Instead, it is either based on the function of the encompassing gene for a mutation to a different amino acid or deletion at the same position. # all three mutations appear together; RBM = receptor binding motif; RBD = receptor binding domain; NTD= N-terminal domain; FCS = S1/S2 furin cleavage site; HR1 = heptad repeat 1; nAbs = neutralizing antibodies.
